# Decreased Dorsomedial Striatum Direct Pathway Neuronal Activity Is Required for Learned Motor Coordination

**DOI:** 10.1523/ENEURO.0169-22.2022

**Published:** 2022-10-10

**Authors:** Stefano Cataldi, Clay Lacefield, N Shashaank, Gautam Kumar, Siham Boumhaouad, David Sulzer

**Affiliations:** 1 Departments of Psychiatry, Columbia University Division of Molecular Therapeutics, New York State Psychiatric Institute, New York, NY 10032; 2 Division of Systems Neuroscience, New York State Psychiatric Institute, New York, NY 10032; 3 Department of Computer Science, Columbia University, Schapiro Center for Engineering and Physical Science Research, New York, NY 10027; 4 New York Genome Center, New York, NY 10013; 5 Neuroscience Program, University of Maryland School of Medicine, Baltimore, MD 21201; 6 Physiology and Physiopathology, Faculty of Sciences, Mohammed V University, Rabat 1014, Morocco; 7 Departments of Neurology, Columbia University, New York, NY 10032; 8 Department of Pharmacology, Columbia University, New York, NY 10032

**Keywords:** direct pathway, dopamine, motor learning, skill acquisition, striatum, treadmill

## Abstract

It has been suggested that the dorsomedial striatum (DMS) is engaged in the early stages of motor learning for goal-directed actions, whereas at later stages, control is transferred to the dorsolateral striatum (DLS), a process that enables learned motor actions to become a skill or habit. It is not known whether these striatal regions are simultaneously active while the expertise is acquired. To address this question, we developed a mouse “Treadmill Training Task” that tracks changes in mouse locomotor coordination during running practice and simultaneously provides a means to measure local neuronal activity using photometry. To measure change in motor coordination over treadmill practice sessions, we used DeepLabCut (DLC) and custom-built code to analyze body position and paw movements. By evaluating improvements in motor coordination during training with simultaneous neuronal calcium activity in the striatum, we found that DMS direct pathway neurons exhibited decreased activity as the mouse gained proficiency at running. In contrast, direct pathway activity in the DLS was similar throughout training. Pharmacological blockade of D1 dopamine receptors in these subregions during task performance demonstrated that dopamine neurotransmission in the direct pathway activity is necessary for efficient motor coordination learning. These results provide new tools to measure changes in fine motor skills with simultaneous recordings of brain activity and reveal fundamental features of the neuronal substrates of motor learning.

## Visual Abstract

**Figure:**
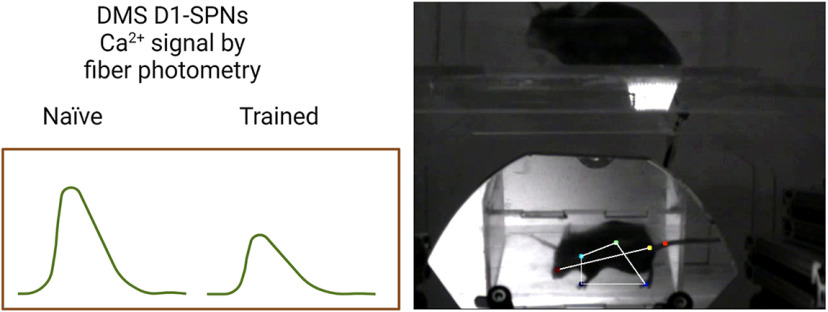

## Significance Statement

Motor disorders, including Parkinson’s disease, present with several motor dysfunction, including degradation of motor learning. While individuals with Parkinson’s disease are able to learn, certain aspects of learning, especially automatic responses to feedback, are damaged. Our current knowledge of motor learning comes from studies on models of motor dysfunction or analysis of simple in-laboratory skills that may not apply to more complex skills. In this work, we developed the “Treadmill Training Task” to track changes in mouse locomotor coordination during practice at running that simultaneously provides a means to measure local neuronal activity using photometry. This work will assist in the development of more efficient therapies for motor impairments because of a multitude of condition, including Parkinson’s disease.

## Introduction

Neural circuits in the striatum are central to basal ganglia functions, including the learning and organization of motor skills. The development of techniques to record the activity of specific populations of neurons in the striatum along with pharmacological or genetic models of motor disorders has revealed new details about the pathways that underlie motor performance and motor learning. The dorsal portion of the striatum has been found to be particularly important for expressing automatic actions (Yin et al., 2009). In rodents, this region is typically separated into two major regions: the dorsomedial striatum (DMS) and the dorsolateral striatum (DLS), corresponding to the human caudate and putamen, respectively. The DMS receives afferents from prefrontal and associative cortices, while the DLS mostly receives input from sensorimotor cortical areas (Graybiel, 2008; Hunnicutt et al., 2016; Cataldi et al., 2022).

*In vivo* electrophysiological recordings indicate that the DMS is engaged in early training on a rotarod (Yin et al., 2009; Lingawi and Balleine, 2012), a task commonly used to study motor coordination, while the DLS is particularly active later in training. A recent study that analyzed corticostriatal synaptic responses in brain slices prepared from mice trained on the rotarod indicated a transient decreased postsynaptic response by DMS striatal spiny projection neurons (SPNs) following training (Badreddine et al., 2022). However, single unit recording during a lever press task showed a similar activity pattern in DLS and DMS after this skill was acquired (Vandaele et al., 2019).

SPNs constitute the majority of striatal neurons (Silberberg and Bolam, 2015) and are generally classified as either D1 dopamine receptor expressing SPNs that form the direct pathway (D1-SPNs) or D2 dopamine receptor expressing SPNs that form the indirect pathway (D2-SPNs; Gerfen et al., 1990; Kawaguchi et al., 1990; Gangarossa et al., 2013; Wall et al., 2013). D1-SPNs axons project to basal ganglia output nuclei, particularly the globus pallidus internal segment and substantia nigra pars reticulata, while D2-SPNs project to the globus pallidus external segment, thus making indirect connection with output nuclei (Albin et al, 1989; DeLong, 1990). According to these classical models, activation of the direct pathway releases neurons in the motor thalamus from inhibition, thus promoting movement. Consistent with these models, optogenetic activation of the D1-SPNs increases locomotion and reduces freezing (Kravitz et al., 2010).

The activity of D1-SPNs has been studied at the level of the initiation, termination, and velocity of a specific motor action (Jin et al, 2014), but there has been little investigation of the role of such circuits during motor learning, an important and dynamic phase of behavioral performance (Cataldi et al., 2022). Computational analysis of behavioral and physiological data during task learning promises to provide a means to elucidate the basis of learning complex motor actions, such as running, under a range of experimental conditions during this crucial phase.

Here, we introduce a paradigm to measure fine changes in motor coordination *in vivo* as a mouse acquires the ability to run on a motorized treadmill while simultaneously recording activity of direct pathway neurons in the DLS or DMS, to study the neural and behavioral changes that occur across early and late stages of learning. We combine a well-established pose tracking method, DeepLabCut (DLC; Mathis et al., 2018), with customized Python-based software to analyze and interpret the behavioral data at different phases of learning. We then investigated changes in calcium activity measured by fiber photometry within striatal regions over training. Our results indicate that DMS activity is reduced over training in running on the treadmill, while DLS activity is similar during early and late stages of learning. Furthermore, pharmacological inhibition of D1-SPNs by D1 antagonists delays skill acquisition during early training in both DMS and DLS, without affecting performance after the task is learned.

## Materials and Methods

### Animals

Wild-type (WT) and transgenic mice expressing Cre-recombinase in D1-expressing SPNs (D1-cre) were used for these experiments. All animal procedures were approved by the Institutional Animal Care and Use Committee of the New York State Psychiatric Institute. Experiments were performed on three- to six-month-old male and female mice. C57BL/6 mice from The Jackson Laboratory (Jax #000664) were used as control animals. D1-Cre transgenic mice were obtained from MMRC/Jax (ey262 D1-cre tg-drdla-cre) and crossed with C57BL/6 mice. Animals were kept on a reversed 12/12 h light/dark cycle (lights are off from 11 A.M. to 11 P.M.) with free access to food and water. Behavior testing occurred during daytime (between 12 and 4 P.M.) and under red light to ensure minimal disturbance of the animals’ sleep cycle. On average animals were housed in groups of two or three per cage. Mice were handled daily for the 3–5 d before experiments to habituate them to the operator.

### Viral expression of GCaMP6f and fiber implants

To achieve neuronal subtype-specific expression of the genetically encoded calcium sensor GCaMP6f in direct pathway SPNs, AAV vectors containing Cre-inducible GCaMP6f (AAV.9.Syn.Flex.GCaMP6f.WPRE.SV40, Addgene; TIter ≥ 2.1 × 13 GC/ml) were injected into the right DLS or DMS by stereotaxic surgery. During the surgery, a small skull craniotomy (1 × 1 mm) above the injection site was opened with a dental drill. A glass pipette attached to a Nanoject II (Drummond Scientific) was filled with the GCaMP6f AAV and lowered to target locations in the dorsomedial or DLS (tip coordinates from bregma: AP −0.5 mm, ML +2.5 DV −3.2 mm for DLS, AP +1.2 mm ML −0.8 mm DV −2.8 mm for DMS). A total volume of 150 nl AAV vector per site was injected over 10 min. The pipette was left in place for five more minutes before removal. A 4-mm-long 300-µm diameter optic fiber (Doric Lenses; MFC 300/370–0.22_4 mm_MF2.5_FLT) was subsequently lowered to the same coordinates. The skull was then covered with dental acrylic to secure the optic fiber in place. Alternatively, opto-fluid cannulas (Doric Lenses; OmFC_MF1.25_300/370-0.22_4.2_FLT_4) were implanted for simultaneous GCaMP6f recording and local microinjection of drug or saline. Animals were allowed to recover, and photometry experiments were performed four weeks after surgery, for optimal viral expression. Three to 5 d before running on the treadmill, animals were placed in an open field chamber to record baseline calcium signals and habituate to the tethered optical fiber.

### Custom-built treadmill

To precisely control the animal’s locomotion, mice were placed on a custom motorized treadmill (Extended Data Fig. 1-1*A*). The treadmill consisted of a 1-m clear belt stretched between two 3 inch diameter acrylic wheels on an aluminum frame (8020.net). Treadmill speed was controlled by an Arduino-based system (OpenMaze.org, OM4 board) that adjusted the speed of a 12 V gear motor attached to the axle of one of the treadmill wheels through pulse-width modulation (PWM). Belt speed was measured using a quadrature rotary encoder (Digikey #ENS1J) attached to the other axle and decoded by the Arduino. The Arduino/OpenMaze setup was also used to send synchronization pulses to coordinate behavior with video recording and fiber photometry. Mouse movement on the treadmill was constrained by placing the mouse into a six-inch-long clear acrylic box over the center of the treadmill and covering the entire width of the belt, which ensured the animal was walking along the belt to avoid being forced into the back wall during belt movement. An acrylic mirror was fixed at a 45° angle under the clear acrylic box and mesh belt to allow high speed videography of the animal’s locomotion from lateral and ventral viewpoints simultaneously.

### Treadmill Training Task

Mice undergoing experimental procedures were weighed before testing. All tests were performed in the morning between 9:30 A.M. and 1:30 P.M., during the animals’ typical awake phase. All animals were assessed at four to six months of age, and all experimentation and analysis were conducted with the experimenter blinded to the location of the implant or condition. After 3-d familiarization to experimenter handling, mice underwent the following training paradigm.

**Figure 1.**
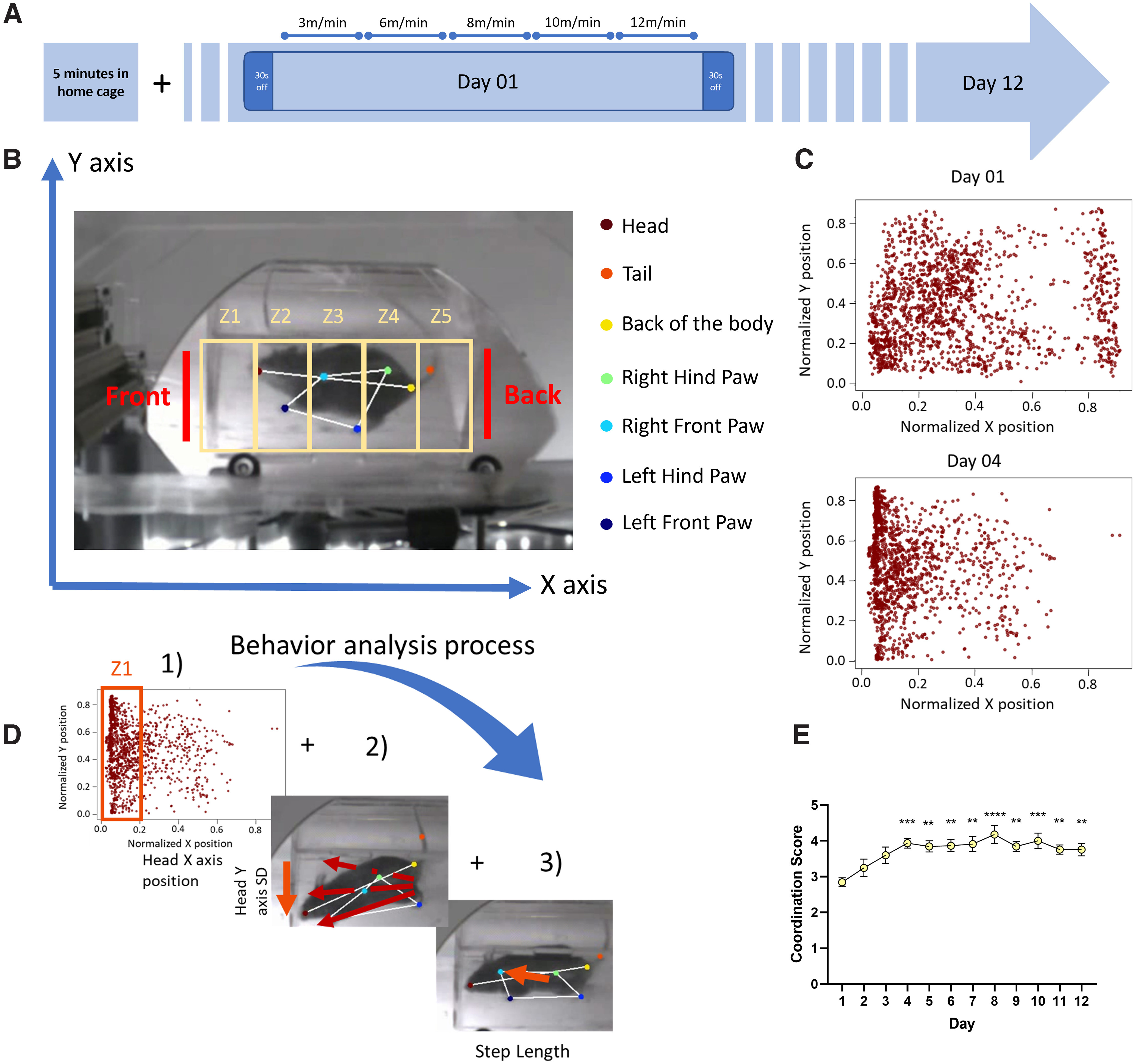
***A***, Timeline of behavioral protocol in the Treadmill Training Task. On each training day, a mouse is connected to the photometry patch cord in its home cage and a 5-min calcium baseline signal recorded. After this habituation period, the mouse is placed on the treadmill and allowed to explore the environment for 30 s. The treadmill motor is then started at a velocity of 3 m/min, with the speed increased every 60 s up to 12 m/min, after which the treadmill is turned off for another 30 s. This protocol is repeated for 12 consecutive days. A photograph of the treadmill is shown in Extended Data Figure 1-1. ***B***, Schematic of the behavioral analysis. Positions of mouse body parts are obtained by analysis with DeepLabCut. For head position, the ventral 2D field of view are divided into five zones and the probability of the head of the mouse to be in each zone calculated. Color code for the body part is indicated in the legend on the right. ***C***, Samples of head positions on day 1 (top) and day 4 (bottom) for a control mouse. Each point represents the head position in one frame and all frames from one video/session are overlapped. On day 1, the head position was frequently toward the rear of the field, indicating the mouse was falling behind the belt speed and often hitting the back wall. By day 4, the animal was able to keep up with the moving belt, and so the position of the head was more consistently toward the front. Plots of the four paws are shown in Extended Data Figure 1-2. ***D***, Process for obtaining the mouse motor coordination score. Motor coordination score is calculated from the expected value of the head position in the five zones, position along the *y*-axis (calculated from standard deviation, SD, of *y*-axis value), and step length (distance between steps for each paw), as detailed in Materials and Methods. ***E***, Motor coordination score for a cohort of control animals. Control mice show significant improvement in the coordination score over the 12 d of testing (*n* = 9; one-way ANOVA *p *<* *0.001, Bonferroni’s multiple comparisons test as detailed in main text). Mouse weight and data by sex is shown in Extended Data Figure 1-1.

Mice were placed on the treadmill within the clear inbuilt open box and were left free to explore the environment for 30 s. After the 30 s, the treadmill movement was activated through the Arduino system at a low speed of 3 m/min, with an increment every 60 s up to 12 m/min (total of five speeds; intermediate speeds are 6, 8, and 10 m/min; Fig. 1*A*). After 5 min of running, the treadmill was turned off for a final baseline recording of 30 s. Finally, animals were removed from the treadmill and returned to their home cage. This process was then repeated for 12 consecutive days, including home cage baseline recording and pre/postrunning treadmill recording epochs. Animal weight was recorded every day after training and showed no significant changes (Extended Data Fig. 1-1*B*). The Arduino source code is freely available on https://github.com/DSulzerLab/Motor_coordination_score and attached as Extended Data 1.

10.1523/ENEURO.0169-22.2022.f1-1 Extended Data Figure 1-1 ***A***, The custom-built treadmill system. A clear circular belt is moved by a motor controlled by a microcontroller. A clear acrylic box is placed around the running area to prevent the mouse from jumping off the treadmill during the experiment. A mirror is placed below the treadmill to visualize movement of all four paws during running via a camera located on the side (not in picture). ***B***, Mouse weight was recorded before each day of experiment; 12 consecutive days of training did not affect mouse weight (one-way ANOVA *p *=* *0.90 for time). Data showing differences between male and female weight at this age (3–4 months of age; *n* = 11 males; *n* = 7 females; two-way ANOVA *p *<* *0.0001). ***C***, Motor coordination score for all cohorts by sex. Male and female animals show similar significant improvement in the coordination score over the 12 d of testing but without any differences between the two sexes (males *n* = 15 and females *n* = 11, two-way ANOVA Day vs Sex *p *=* *0.33, Day ****p <* *0.0001, Sex *p *=* *0.07, Subject ****p <* *0.0001). Download Figure 1-1, TIF file.

**Figure 2.**
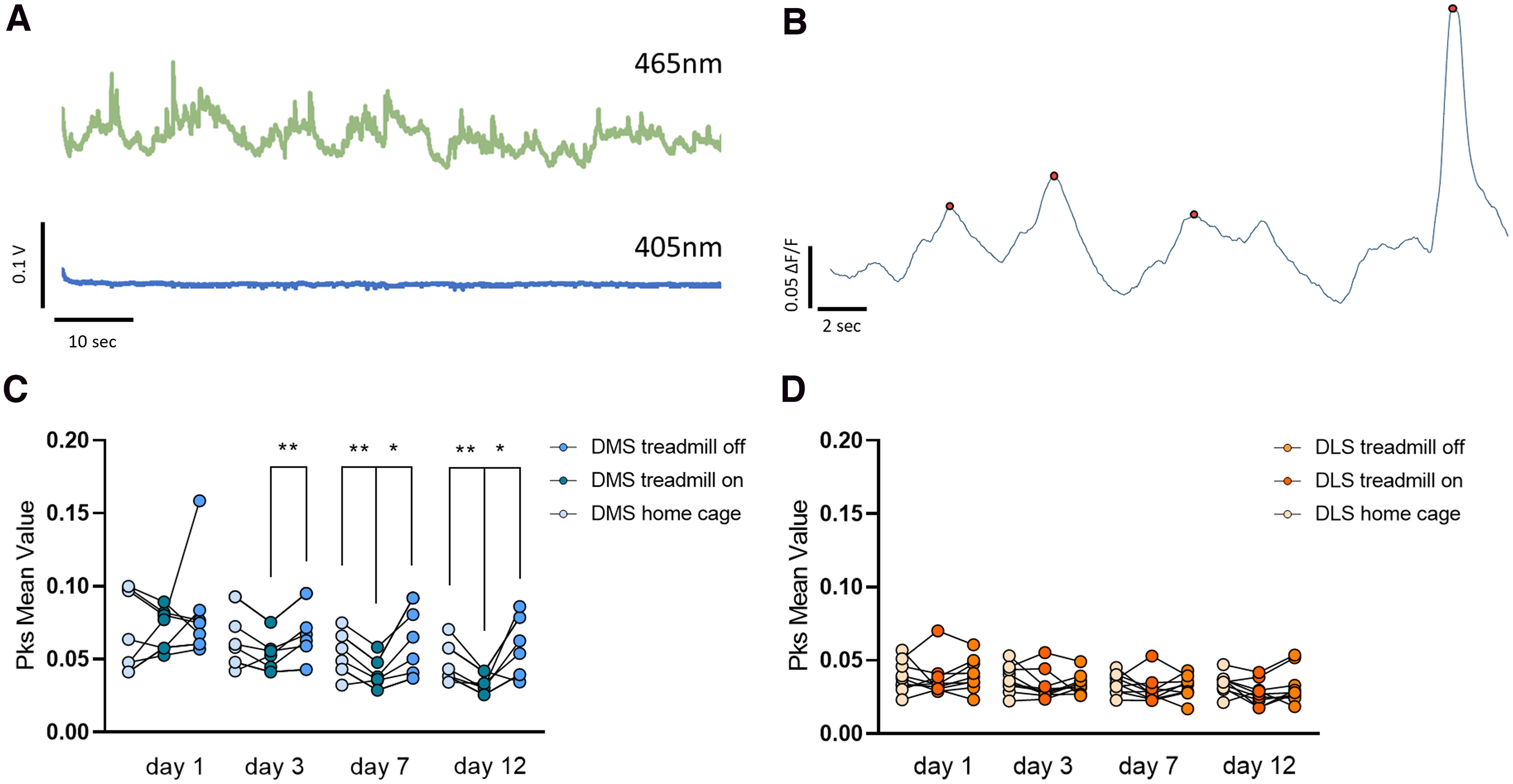
***A***, Raw sample fluorescence traces recorded during baseline with excitation at 405 nm (bottom trace), GCaMP6f isosbestic point, in comparison to calcium signal at 465 nm (top). ***B***, Analyzed sample trace of D1-SPNs calcium signals recorded in the DMS of a mouse running on the Treadmill Training Task. Detected calcium events are indicated by red dots. Extended Data Figure 2-2 shows averaged calcium traces corresponding to changes in the treadmill acceleration (Extended Data Fig. 2-2*A–D*) and during important changes in head position (Extended Data Fig. 2-2*E*,*F*). ***C***, Average event amplitude from treadmill recording. Running epochs at all treadmill speeds are averaged together and referred to as “on time.” The 30 s before running and after the treadmill is turned off are averaged together and referred to as “off time.” Day 1, day 3, day 7, and day 12 are shown. All other days are shown in Extended Data Figure 2-1. DMS D1-SPNs activity during on-time is comparable to off-time and home cage baseline on day 1 (*n* = 7; one-way ANOVA *p *=* *0.66) and becomes significantly lower than off-time activity starting on day 3 (RM one-way ANOVA *p *<* *0.05, Bonferroni’s multiple comparisons test *p <* *0.05 DMS on-time vs DMS off-time), and then on day 7 (RM one-way ANOVA *p *<* *0.01, Bonferroni’s multiple comparisons test *p <* *0.05 DMS on-time vs DMS off-time and *p <* *0.01 DMS on-time vs DMS home cage baseline) and day 12 (RM one-way ANOVA *p *<* *0.05, Bonferroni’s multiple comparisons test *p <* *0.05 DMS on-time vs DMS off-time and *p <* *0.01 DMS on-time vs DMS home cage baseline), suggesting that activity in this region is lower once the skill is acquired. ***D***, Average event amplitude from treadmill recording in the DLS. There are no changes between on and off time for D1-SPNs recorded from the DLS (*n* = 9; day 1: one-way ANOVA *p *=* *0.73; day 3: one-way ANOVA *p *=* *0.34; day 7: one-way ANOVA *p *=* *0.53; day 12: one-way ANOVA *p *=* *0.50). All days and event counts are shown in Extended Data Figure 2-1. Sample images showing site of GCaMP6f injection and optic fiber implants are shown in Extended Data Figure 2-3.

10.1523/ENEURO.0169-22.2022.f1-2 Extended Data Figure 1-2 Heatmaps of paw placement data obtained from DeepLabCut for one mouse on day 1 (***B***) and day 7 (***C***) of the Treadmill Training Task. Each heatmap represents data from a different paw and is color labeled as per the schematic in ***A*** and in Figure 1*B* (top left for the right front paw, light blue dot in panel ***A***; bottom left for the left front paw, dark blue dot in panel ***A***; top right for the right hind paw, green dot in panel ***A***; bottom right for the left hind paw, blue dot for panel ***A***). *x*- and *y*-axes values obtained from tracking are normalized. Each heat map shows likelihood of placing a specific paw in a specific set of coordinates. The blue color represents regions with low number of paw placements, while yellow indicates high chance of placing in those coordinates. ***B***, Heatmaps for one control mouse on day 1 of training. The spread of paw placement indicates poor ability of the mouse to run into place. While running the mouse was placing its paws all over the 2D field and rarely in the same spot. ***C***, Heatmaps for the same control animal on day 7 of training. This data show more consistent paw placement with the mouse stepping mostly in the same area. This shows the ability of the mouse to run in place, indicating improved proficiency in running. Download Figure 1-2, TIF file.

10.1523/ENEURO.0169-22.2022.ed1 Extended Data 1 Custom-made python codes as described in Materials and Methods. Step length analysis: DeepLabCut paw position coordinates are used to calculate the length of each step made by the mouse during running on the treadmill. Mouse metrics: metrics as described in Materials and Methods are obtained from DeepLabCut and normalized within the dimension of the box in which the mouse is running. Coordination score: mouse metrics are combined to obtain the coordination score. Acceleration analysis: time of treadmill acceleration is identified and values from the corresponding calcium trace are extracted and plotted separately. Single event analysis: code identifies significant changes in the values for one of the metrics and extracts the corresponding calcium trace. Download Extended Data 1, ZIP file.

### Photometry recording

Calcium signals were recorded using a commercial fiber photometry apparatus (Doric Lenses). The system consisted of a console connected to a computer, a four-channel programmable LED driver, and two LEDs at 405 and 465 nm connected to fluorescence dichroic mini cubes and photometric multipliers tubes (PMTs). The 405-nm wavelength is the isosbestic point for GCaMP, where fluorescence does not change depending on cytosolic calcium concentration (Extended Data Fig. 2-1*A*). Detection at this wavelength was used to remove background noise (movement artifact or GCaMP auto-fluorescence). The 465-nm excitation provides detection of GCaMP signal where the intensity of fluorescence is proportional to cytosolic calcium concentration.

Calcium signals from each animal were recorded for 5 min as the mice explored their home cage before treadmill testing. This is used as a baseline to evaluate whether there is any loss of signal over the several days of testing, as well as to habituate the animal to the optical cable.

### Calcium signal analysis

Calcium signals were processed using custom-built Python code to remove background noise and detect individual calcium peak events. The process consisted of (1) down sampling the signal to 30 samples/s to match the sampling rate of behavioral recording, (2) normalization of the data and removal of background noise, (3) identification and quantification of peak events (events count and event amplitude). In detail, 465 and 405 signals were normalized using the following formula:

$$\frac{F-\Delta F}{\Delta F},$$

where ΔF is calculated by *a roolling* mean of 125 of the overall trace. Then the 405 signal is subtracted from the 465 signal to remove movement artifacts and background noise. The resulting normalized trace is then analyzed using the Python function *find_peaks*, which identifies individual peak events that have a prominence (actual peak height) greater than 0.02, and are at least 60 samples away from each other (2 s). Individual traces are inspected to ensure accuracy of peak identification. The source code is freely available on https://github.com/DSulzerLab/Calcium_peaks_analysis and attached as Extended Data 1.

We normalized behavior measures between a range of 0–1 and identified events in which the rolling mean varies by 0.1, indicating a significant change in the given measures. Variation in the head position was used to identify events in which the mouse did not keep up with the running tape and hit the back of the box. Variation in step length was used to detect events in which the mouse made a longer step to catch up with the running tape. Corresponding calcium traces were averaged to calculate the likelihood of a calcium peak event to occur at the same time as a given behavioral event. The source code is freely available on https://github.com/DSulzerLab/Motor_coordination_score and attached as Extended Data 1.

### Pharmacological experiments

SCH39166 (Tocris), a selective D1-antagonist, was dissolved in 100% DMSO and then diluted to 1% DMSO concentration with saline (0.9% NaCl) containing 1 mm rhodamine (Rhodamine B Base; Aldrich 234141-10G), for a final concentration of 48.13 μm SCH39166 (total injected 5.7 ng). To ensure that the drug’s effect was not because of the injection or presence of rhodamine, an additional cohort of control animals was injected with the same concentration of DMSO. Saline solution without the drug was mixed with rhodamine to obtain a final concentration of 1% DMSO (sham solution).

The mice were injected locally in the DMS or DLS with 300 nl of either the SCH39166 or control solution, at a rate of 100 nl/min using a microinjection pump (UltraMicroPump III, World Precision Instruments) through an injection cannula attached to the photometry fiber (Doric Lenses, Fl_OmFC_ZF_100/170_4.2) while running on the treadmill. Local injection was confirmed by recording the 565 nm signal from the rhodamine dye (Extended Data Fig. 3-1).

**Figure 3.**
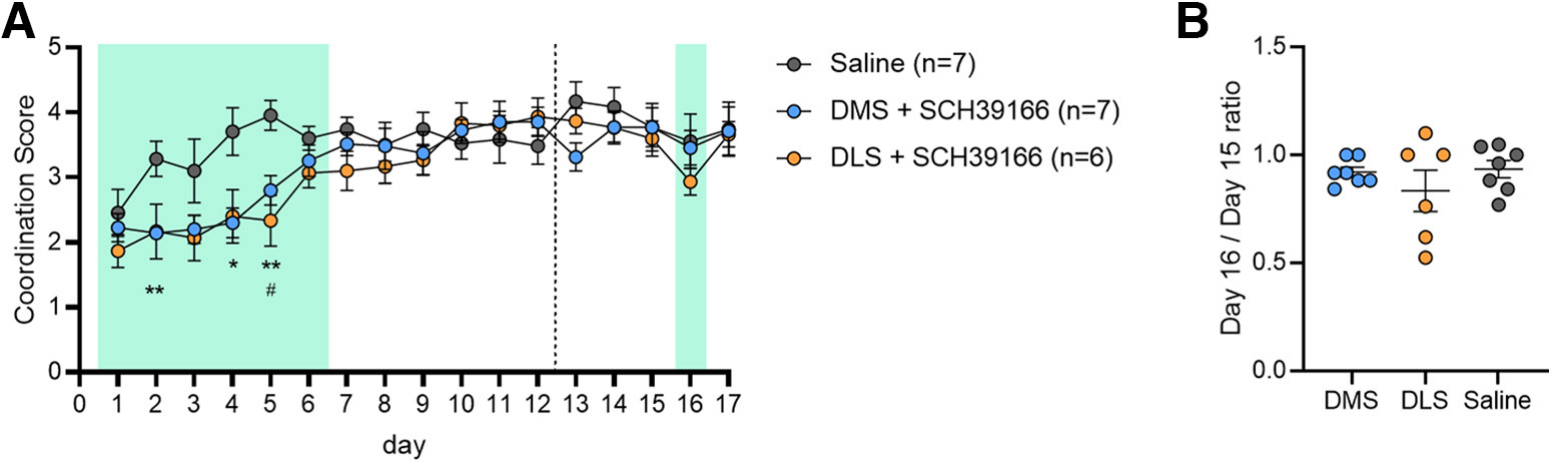
Local D1 neuron inactivation in DMS or DLS during the Treadmill Training Task. Mice were locally injected with a solution of SCH39166, a D1-antagonist. A sample calcium trace after injection is shown in Extended Data Figure 3-1. All animals were injected for the first 6 d of the task (injection running for the first 3 min of the test each day). No drug was injected from day 7 to day 12. Mice were then left to rest for 7 d and tested again for five consecutive days (day 13 to day 17). Another injection of SCH39166 was administered on day 16. Injection days are indicated by green shading. ***A***, D1 antagonism in the DMS caused delayed improvement in the performance, as shown by low coordination score from day 2 to day 5 when compared with saline injected animals (two-way ANOVA Time × Column factor *p *<* *0.05, Bonferroni’s multiple comparisons test for day 2 **p <* *0.01, day 4 *p *<* *0.05, day 5 **p <* *0.01; saline-injected animals received infusion in either the DMS or the DLS and the data were combined). Similar to DMS-injected animals, application of the same D1 antagonist to the DLS altered performance, with a delayed improvement of the coordination score particularly on day 5 (two-way ANOVA Time × Column factor *p *<* *0.05, Bonferroni’s multiple comparisons test for day 5 #*p *<* *0.05). ***B***, After a week without treadmill, both DLS and DMS-injected animals performed similarly to the previous running day. Conversely to DMS-injected mice, DLS-injected animals showed a decrease in performance after a single injection on day 16 (one-way ANOVA *p* < 0.01, Bonferroni’s multiple comparisons test *p* = 0.47), but quickly recovered the following day with no injection. Injection of SCH39166 in the DMS on day 16 did not affect performance as compared with control animals (one-way ANOVA *p* = 0.85).

10.1523/ENEURO.0169-22.2022.f3-1 Extended Data Figure 3-1 ***A***, Representative calcium trace (in blue, detected events indicated by orange dots) of D1-SPNs GCaMP6f signal in the DMS of a freely moving mouse, while injected with a control solution of saline + rhodamine. Rhodamine signal is recorded at 560 nm to confirm injection local to the photometry fiber (red). Local injection did not alter calcium signals in this control. Injection time indicated by arrows. ***B***, Similar recording of D1-SPNs in the DMS during injection of a D1-antagonist (SCH39166, 48.13 µm), in solution with rhodamine. Injection of the antagonist significantly reduced the baseline calcium signal and eliminated calcium events in the DMS. Download Figure 3-1, TIF file.

### Video analysis

Behavioral experiments were recorded using a high speed USB webcam (Sony PS3eye) and commercial video acquisition software (Kinovea), synchronized to the Arduino/OpenMaze behavior system with an infrared LED, and analyzed *post hoc* using DeepLabCut (Mathis et al., 2018) to track movement of individual body parts (the four paws, head, rear of the body, and tail were labeled as shown in Fig. 1 and Movie 1). Data were processed using Google Colaboratory and ∼30,000–40,000 iterations were sufficient for good quality tracking. A sample of an analyzed video is shown (see Movie 1).

Movie 1. Sample video of a D1-cre mouse injected with GCaMP6f in the DSM. Video shows mouse’s body parts labeled and analyzed with DeepLabCut with simultaneous calcium recording. 10.1523/ENEURO.0169-22.2022.video.1

The DeepLabCut results were subsequently processed with custom-built Python code to return a “motor coordination score” from 1 to 5, with 1 representing poor and 5 representing excellent coordination. The coordination score was computed using three values: the expected value of the mouse head’s *x*-axis position, the SD of the mouse head’s *y*-axis position, and the average step length of the four individual paws. First, the expected value of the mouse head’s *x*-axis position was computed by dividing the box into five zones, with zone 1 being the frontmost and zone 5 the rearmost (Fig. 1*B*), and calculating the probability of the head being positioned in a specific zone. Second, the SD of the mouse head’s *y*-axis position was computed to identify the sideways movement. Third, the step length was computed using the Python package “traja” (Shenk et al., 2021), which uses spatiotemporal animal tracking data to estimate parameters such as the length of each step, straightness (running along a straight line), and speed. The average step length for each paw was then computed, and the “paw stability” determined by calculating the step length’s distance from the median of the average step length measurements. Along with the paw stability, the percentiles of the expected values and the *y*-axis SD were computed, and these three values were given weights of 20/40/40, respectively. The percentiles of the weighted average were scaled between 1 and 5 to determine an overall coordination score. We arbitrarily chose the head position for this analysis, although position of either paw can also be used. Parallel analysis of the likelihood of paw placement over training demonstrates a higher fraction of steps within specific coordinates that are closer to the front of the box (Extended Data Fig. 1-2).

Manual scoring of the videos under blinded conditions confirmed the validity and reproducibility of the test and analysis method. The source code is freely available on https://github.com/DSulzerLab/Motor_coordination_score and attached as Extended Data 1.

### Immunohistochemistry

After the completion of the behavioral experiments, mice were terminally anesthetized (euthasol 240 mg/kg, 200 µl, i.p.) and intracardially perfused with PBS then 4% paraformaldehyde (PFA). Brains were extracted and postfixed overnight (4% PFA, 4°C). Coronal slices (100 µm) were obtained by vibratome (Leica VT 1200). Sections were rinsed with 0.6% Triton X-100 in 1× PBS (PBST; 6 × 60 min) and blocked in 10% normal donkey serum (NDS) in PBST [60 min, room temperature (RT)]. Primary antibodies: chicken polyclonal GFP (ab13970 Abcam; 1:500) and rabbit polyclonal anti-tyrosine hydroxylase (TH; ab152 Abcam; 1:500), were applied in 2% NDS in PBST (48 h 4°C) before washing (6 × 60 min PBST) and secondary incubation with species specific Alexafluor IgG secondary antibodies (60 min, RT, Invitrogen; 1:500). Tissues were washed again in 0.1% PBST (6 × 60 min), then mounted using DAPI Fluoromount-G (0100–20, SouthernBiotech). Images were acquired using a 20× oil objective on an Olympus microscope (see sample images in Extended Data Fig. 2-3).

### Code accessibility and statistics and data reporting

The code described in the paper is freely available online at https://github.com/DSulzerLab. The code is available as Extended Data 1.

Data are presented throughout as mean ± SEM, where *n* is the number of animals. Comparisons were conducted by one-way or two-way ANOVA with appropriate *post hoc* tests detailed in the text, using Prism 9.0 (GraphPad).

## Results

### Treadmill running

To explore motor coordination learning during locomotion, we designed a custom-made motorized treadmill that provides detection of fine improvement in paw mobility as a mouse learns to run on the treadmill at a range of velocities (referred as the Treadmill Training Task; Fig. 1*B*; Extended Data Fig. 1-1*A*).

A cohort of WT mice were initially examined. On the first day of the protocol, animals were placed on the treadmill and allowed to freely explore the environment for 30 s, after which the treadmill was activated using the Arduino system. After 5 min of running, the treadmill was turned off for a final baseline recording of 30 s (Fig. 1*A*). This protocol was repeated for 12 consecutive days to track changes in the mouse running motor coordination.

The videos were analyzed with the machine-learning based videographic analysis platform DeepLabCut (DLC; Mathis et al., 2018), which allowed us to track paw movements in space and time. DLC provides *x* and *y* coordinates of the 2D plane of the video for each body part of interest across individual frames (Fig. 1*B*,*C*; Extended Data Fig. 1-2). Results obtained by DLC were processed through custom-built Python scripts to compute a “motor coordination score” (see Materials and Methods) using variables such as the mouse head’s position within the box zones (*X*-axis), the mouse’s sideways movement along the treadmill mouse’s sideways movement along the treadmill (y-axis standard deviation, SD), and the mouse paw coordination on the treadmill (average step length). Individually, each variable did not accurately represent the running capability. For example, at lower speeds, running requires little effort for the animals and they often explore the box and move sideways, rear, or groom. This would affect a coordination score based only on the length of each step (data not shown). Similarly, at higher speed, the mouse is often mostly in the rear section of the box as it tries to catch up with the moving tape, yielding a low score for the head position. We find that a combination of these factors provides a more accurate estimate of coordination. Indeed, in the initial days of training, mice had a significantly lower motor coordination score than during later sessions (Fig. 1*E*), as they had not been exposed to the treadmill before and displayed poor coordination during running: the task naive mice would tend to fall behind and make longer steps trying to catch up with the running tape. Moreover, naive animals tended to run sideways rather than along a straight line (Extended Data Fig. 1-2*B*). On later days of training, as mice accrued competency in running and better coordination, their score improved (Fig. 1*E*) and their likelihood of stepping in the same region increased (Extended Data Fig. 1-2*B*). Control animals trained on the treadmill showed a significantly higher coordination score within the fourth day of training. By day 4, all mice performed significantly better than on the first training day (one-way ANOVA *F*_(11,96)_ = 3.753, *p *<* *0.001, Bonferroni’s multiple comparisons test day 4 ***p <* *0.001, day 5 **p <* *0.01, day 6 **p <* *0.01, day 7 **p <* *0.01, day 8 ****p <* *0.0001, day 9 **p <* *0.01, day 10 ***p <* *0.001, day 11 **p <* *0.01, day 12 **p <* *0.01; Fig. 1*E*), with no significant differences between male and females mice (two-way ANOVA Day vs Sex *F*_(11,264)_ = 1.134, *p *=* *0.33, Day *F*_(6036,144.9)_ = 11.61, *p *<* *0.0001, Sex *F*_(1,24)_ = 3.502, *p *=* *0.07, Subject *F*_(24,264)_ = 7.96, *p *<* *0.0001; Extended Data Fig. 1-1*C*).

### DMS and DLS D1-SPNs calcium activity during running

We next explored the role of the direct pathway in skill acquisition by recording calcium signals from D1-SPNs in either the DMS or DLS. Recording of baseline D1-SPNs activity in the DMS show no significant changes over the 12 d (one-way ANOVA *F*_(11,84)_ = 1.562, *p *=* *0.13; Extended Data Fig. 2-1*C*). Similarly, no changes were found in D1-SPNs activity in the DLS (one-way ANOVA *F*_(11,96)_ = 0.61, *p *=* *0.82; Extended Data Fig. 2-1*D*).

During treadmill running, GCaMP6f-injected mice performance was identical to control animals (one-way ANOVA for DMS mice *F*_(11,84)_ = 2.547, *p *<* *0.01, Bonferroni’s multiple comparisons test day 4 *p <* *0.05, day 5 **p <* *0.05, day 6 *p <* *0.05, day 7 **p <* *0.01, day 8 **p <* *0.01, day 9 *p <* *0.05, day 10 **p <* *0.01, day 11 **p <* *0.01, day 12 *p <* *0.05 and for DLS mice *F*_(11,96)_ = 2.547, *p *<* *0.05, Bonferroni’s multiple comparisons test day 6 #*p *<* *0.05, day 7 #*p *<* *0.05, day 8 ##*p *<* *0.01, day 9 #*p *<* *0.05, day 10 #*p *<* *0.05, day 11 #*p *<* *0.05, day 12 ##*p *<* *0.01; Extended Data Fig. 2-1*B*), indicating that the surgery, viral expression, and implant did not affect the ability to run on the treadmill. We then evaluated brain activity with the coordination score to examine neural changes as the mice gained proficiency at running on the motorized treadmill. No difference in behavior or calcium signals was found during the 30-s periods before and after the running time (data not shown), and the data from both off periods were merged and reported as “off-time.”

On day 1 of training, all mice showed similar average calcium event amplitude between baseline, the running (on-time; independently of the speed of the treadmill) and pre/postrunning epochs in which the treadmill was off (off-time; Fig. 2*C* for DMS RM one-way ANOVA *F*_(2,8)_ = 0.662, *p *=* *0.66; Fig. 2*D* for DLS RM one-way ANOVA *F*_(2,12)_ = 0.258, *p *=* *0.73). Similarly, event rates were comparable between baseline recording and while on the treadmill, whether it was on or off (three conditions analyzed separately over the 12 d: baseline calcium, on-time, and off-time, Extended Data Fig. 2-1*E*; one-way ANOVA for DMS baseline *F*_(11,84)_ = 1.761, *p *=* *0.08, for DMS time-on *F*_(11,84)_ = 1.971, *p *=* *0.07, for DMS time-off *F*_(11,84)_ = 1.184, *p *=* *0.31; Extended Data Fig. 2-1*F*; one-way ANOVA for DLS baseline *F*_(11,96)_ = 0.767, *p *=* *0.67, for DLS time-on *F*_(11,96)_ = 0.849, *p *=* *0.59, for DLS time-off *F*_(11,96)_ = 0.447, *p *=* *0.93).

10.1523/ENEURO.0169-22.2022.f2-1 Extended Data Figure 2-1 ***A***, Sample image from DeepLabCut as the mouse is running on the treadmill. Each paw, the head, the base of the tail, and the tail are labeled by colored dots. ***B***, Coordination score from treadmill running for DMS-injected and DLS-injected animals. Mice performed significantly better within 2–4 d from day 1 of training, indicating that surgery, viral expression, and optic fiber implant did not affect running abilities and motor learning (one-way ANOVA **p <* *0.01 for DMS mice and *p <* *0.05 for DLS mice). ***C***, ***D***, Average calcium event peak amplitudes for D1-SPNs in the DMS (***C***) and DLS (***D***) over the 12 d of training. Peak epoch amplitudes are compared with the baseline home cage recording. There is no significant change in home cage baseline (one-way ANOVA *p *=* *0.13 for DMS and *p *=* *0.73 for DLS) and time-off (one-way ANOVA *p *=* *0.21 for DMS and *p *=* *0.50 for DLS). Average peak amplitude for DMS D1-SPNs significantly decreased during running time (one-way ANOVA *p *<* *0.0001 for on-time alone, * represents *post hoc* significant values, and RM two-way ANOVA *p *<* *0.01 when comparing on-time to off-time, # represents *post hoc* significant values; detailed statistics in the main text) although peak amplitudes average levels remained unchanged for DLS D1-SPNs (one-way ANOVA *p *=* *0.64). ***E***, ***F***, Average calcium event rate for D1-SPNs in the DMS (***E***) and DLS (***F***). Measurements for epochs of running time (treadmill on), time in which the treadmill is off, and event counts in home cage baseline recording were overlapped. There were no significant changes in the average event rate over the 12 d of training for DMS (one-way ANOVA *p *=* *0.08 for home cage baseline recording, *p *=* *0.07 for running time, and *p* = 0.31) or DLS recording (one-way ANOVA *p *=* *0.67 for home cage baseline recording, *p *=* *0.59 for running time, and *p* = 0.93). Download Figure 2-1, TIF file.

10.1523/ENEURO.0169-22.2022.f2-2 Extended Data Figure 2-2 ***A***, ***B***, Sample average calcium traces from D1-SPNs in the DMS of one mouse at the time of which the treadmill is turned on (indicated by dashed black line) for day 1 (***A***) and for day 12 (***B***). There is no apparent increase in calcium activity at that time. ***C***, ***D***, Similarly, there is no obvious increase in activity at the time in which the treadmill switches to the highest speed (10 m/min) for both day 1 (***C***) and day 12 (***D***; time of acceleration indicated by dashed black line). ***E***, ***F***, Sample average calcium traces for the DMS of one mouse at the time of significant events [high variation in mouse head position (***E***) or in step length (***F***)]. Mouse head position and step length were analyzed to detect moments during running in which the value significantly differs from the average. Corresponding calcium traces were identified and averaged together. No significant peak in calcium activity was detected during these events. Download Figure 2-2, TIF file.

10.1523/ENEURO.0169-22.2022.f2-3 Extended Data Figure 2-3 ***A***, Schematic of location of optic fibers for mice implanted in the DMS (left) and the DLS (right). ***B***, Sample micrographs of coronal sections of the dorsal striatum of D1-cre animals injected with AAV vectors containing Flex-GCaMP6f. GCaMP6f expression is confirmed by GFP immunolabeling (yellow) co-stained with TH antibody (blue) to visualize the striatum. Download Figure 2-3, TIF file.

Interestingly, DMS D1-SPNs calcium signals displayed a significant decrease in overall event amplitude beginning on day 3 while running (on-time) compared with off-time levels (RM one-way ANOVA *F*_(2,8)_ = 6.169, *p <* *0.05, Bonferroni’s multiple comparisons test *p <* *0.05 DMS on-time vs DMS off-time; Fig. 2*C*), which remained consistent throughout the 12 d of testing (for day 7 RM one-way ANOVA *F*_(2,8)_ = 12.53, *p *<* *0.01, Bonferroni’s multiple comparisons test *p <* *0.05 DMS on-time vs DMS off-time and **p <* *0.01 DMS on-time vs DMS home cage; for day 12 RM one-way ANOVA *F*_(2,8)_ = 7.618, *p *<* *0.05, Bonferroni’s multiple comparisons test *p <* *0.05 DMS on-time vs DMS off-time and **p <* *0.01 DMS on-time vs DMS home cage; Fig. 2*C*). The difference is also apparent when peak epochs amplitudes are compared with the baseline home cage recording (for DMS on-time one-way ANOVA *F*_(11,84)_ = 0.887, *p *<* *0.0001, Bonferroni’s multiple comparisons test: day 4 *p <* *0.05, day 5 ***p <* *0.001, day 6 ***p <* *0.001, day 7 ****p <* *0.0001, day 8 ****p <* *0.0001, day 9 ****p <* *0.0001, day 10 ****p <* *0.0001, day 11 ****p <* *0.0001, day 12 ****p <* *0.0001; Extended Data Fig. 2-1*C*), while off-time average peak amplitudes do not change over training (for DMS off-time one-way ANOVA *F*_(11,84)_ = 1.349, *p *=* *0.21; Extended Data Fig. 2-1*C*). The data remain significant when on-time is compared directly to off-time recording (RM two-way ANOVA where the two conditions are on-time vs off-time, Day × Condition *F*_(11,132)_ = 1.045, *p *=* *0.41, Day *F*_2.682,32.18_ = 7.864, *p *<* *0.001, Condition *F*_(1,12)_ = 7.093, *p *<* *0.01, Subjects *F*_(12,132)_ = 8.244, *p *<* *0.0001, Bonferroni’s multiple comparisons test for day 7 #*p *<* *0.05, day 8 ##*p *<* *0.01, day 9 #*p *<* *0.05, day 10 #*p *<* *0.01, day 11 ##*p *<* *0.01, day 12 ##*p *<* *0.01; Extended Data Fig. 2-1*C*). Conversely, D1-SPN calcium signals recorded from the DLS showed no significant difference in either calcium event amplitude or rate between running time and off-time over training (Fig. 2*D*; day 3: RM one-way ANOVA *F*_(2,12)_ = 1.150, *p *=* *0.34; day 7 RM one-way ANOVA *F*_(2,12)_ = 0.631, *p *=* *0.53; day 12: RM one-way ANOVA *F*_(2,12)_ = 3.235, *p *=* *0.07; Extended Data Fig. 2-1*D*, for DLS on-time one-way ANOVA *F*_(11,96)_ = 0.801, *p *=* *0.64, for DLS off-time one-way ANOVA *F*_(11,96)_ = 0.947, *p *=* *0.50).

When the data were further analyzed by motor feature (step length, head position) there was no apparent correlation between each specific behavior events and calcium epochs. Specifically, no apparent peak was present at times in which the mouse fell behind and attempted to catch up with the moving tape (evaluated as a sudden change in the head position; Extended Data Fig. 2-2). Similarly, there was no apparent increase in calcium signals at events in which the mouse performed larger steps to position itself in the front of the cage after struggling to keep up (data not shown).

Because we increased the speed of the treadmill every minute, we investigated whether there was an increase in calcium activity at the time of each acceleration. We did not observe a clear peak event at the specific time any of the acceleration on any of the 12 d of training (example traces in Extended Data Fig. 2-2).

### Inhibition of D1-SPNs with a D1-receptor antagonist

Despite the absence of calcium peaks associated with specific motor features of running behavior, we hypothesized that the overall change in DMS activity during training may be important for skill acquisition. To investigate this, we locally injected SCH39166, a selective D1-antagonist, into the DMS or DLS as the mouse was running to inhibit the activity of direct pathway D1-SPNs during task learning. An example trace from a DMS implanted mouse shows significant reduction in calcium signals in D1-SPNs as the drug is injected, while the red wavelength rhodamine signal increased (Extended Data Fig. 3-1), confirming local infusion. A cohort of mice was injected with a saline solution in either the DMS or the DLS (data combined) as a control for possible effect of the injection.

All SCH39166-injected mice, independent of the region of injection, showed delayed improvement in performance until day 5. By day 6, performance quickly improved (Fig. 3*A*). The coordination scores for days 2 through 5 were significantly lower for animals injected with SCH39166 in the DMS (two conditions: SCH39166 vs saline, two-way ANOVA Day × Condition *F*_(16,192)_ = 2.019, *p *<* *0.05, Day *F*_5.134,61.61_ = 5.762, *p *<* *0.001, Condition *F*_(1,12)_ = 3.695, *p *=* *0.08, Subjects *F*_(12,192)_ = 4.374, *p *<* *0.0001, Bonferroni’s multiple comparisons test for day 2 **p <* *0.01, day 4 *p <* *0.05, day 5 **p <* *0.01), than for saline-injected animals. Interestingly, injections of the D1-antagonist in the DLS also slowed learning, though this only reached significance for day 5 (two conditions: SCH39166 vs saline, two-way ANOVA Day × Condition *F*_(16,176)_ = 1.943, *p *<* *0.05, Day *F*_5.887,64.76_ = 6.037, *p *<* *0.0001, Condition *F*_(1,11)_ = 5.476, *p *<* *0.05, Subjects *F*_(11,176)_ = 4.422, *p *<* *0.0001, Bonferroni’s multiple comparisons test for day 5 #*p* < 0.05).

To investigate DMS and DLS contributions to performance once the skill is acquired, we recorded 5 additional days one week after the first session (labeled day 13 to day 17). On day 16, mice were injected with the same dose of SCH39166 as previously. Interestingly, injection in the DLS decreased the coordination score, although this did not reach significance by *post hoc* test (one-way ANOVA *p* < 0.01, Bonferroni’s multiple comparisons test *p* = 0.47; Fig. 3*A*,*B*). No decrease was observed on the following day when mice were tested again without the D1-antagonist. D1-receptor inhibition in the DMS on day 16 resulted in no impairment in performance (one-way ANOVA *p* = 0.85; Fig. 3*A*,*B*). The lack of change in coordination score on day 16 for DMS injected mice suggest that D1 dopamine receptor activation in this region is highly relevant in early training but is less necessary for performance of the previously learned task.

## Discussion

Current knowledge of the synaptic circuitry underlying motor learning comes from studies analyzing simple tasks, such as lever presses to achieve a food or drug reward or counting the number of falls from a motorized rotarod. While much has been learned from these approaches, they may not be applicable to learning more complex skills, such as changes in motor coordination during practice, a feature of activities ranging from athletic tasks to performance on a musical instrument. The training protocol introduced here, referred as the Treadmill Training Task, provides a means to record activity of the direct pathway in the medial and lateral portions of the dorsal striatum while analyzing complex motor activity in freely moving animals in a carefully controlled locomotor task. We furthermore developed a computational method to interpret multi-limb behavioral data and identify patterns of motor function that contribute to the improvement of coordination of a running mouse during practice. In doing so, we found that DMS and DLS activity progresses differently as animals gain skill in a motor task, revealing neuroanatomic specificity in progressive motor learning.

Our results are consistent with the hypothesis that DMS activity might decrease during complex motor learning (Cataldi et al., 2022), and is consistent with reports by Kupfershmidt and colleagues showing that activity of associative inputs to the DMS are active during skill acquisition but less so during the execution of a mastered skill (Kupferschmidt et al., 2017). Similarly, a recent investigation shows that corticostriatal postsynaptic responses in the DMS undergo a transient decrease after training on the rotarod task, although those recordings were conducted in brain slice preparations and could not measure changes in activity during the task training (Badreddine et al., 2022).

Although we found that the amplitude of calcium events in the DMS changes over training, the rate of calcium activity transients did not. Our results are consistent with a report by Wightman et al., that demonstrated distinct active and silent regions in the striatum that exhibit different SPN activity and dopamine release patterns as a task is acquired (Owesson-White et al., 2016), a feature further consistent with the recent study on brain slices from trained animals (Badreddine et al., 2022). Badreddine’s study suggests that a reorganization of high activity cells increases the signal-to-noise ratio and contributes to better transmission of information. In this regard, we note that calcium signals detected by fiber photometry describe the bulk activity of a multitude of neurons, and an overall reduction in amplitude but not in the number of events could indicate that fewer, “specialized” neurons fire at the same rate once the skill is acquired (Bamford et al., 2018). Future experiments may explore this hypothesis by examining single cell activity over training using imaging techniques that use miniature head-mounted microscopes.

An important advantage to our approach is the ability to expose the regions being recorded to local pharmacological manipulations. Such experiments demonstrate that inhibition of D1 receptors delayed motor coordination learning in both the DMS and the DLS. The ability of mice to still learn improved performance on the Treadmill Training Task, albeit with a substantial lag compared with saline-treated mice, suggests that additional regions or cell types may promote motor learning with a lower efficiency in the absence of striatal activity. Interestingly, while no change in DLS activity was detected during training, inhibition of D1 dopamine receptors in the DLS delayed learning to an extent similar to the inhibition of DMS D1-SPNs. Similarly, Badreddine and colleagues did not find changes in mean amplitude nor overall changes in percentage of high activity cells in DLS brain slices because of training, although they detected a spatial reorganization of clusters of high activity cells in this region in late training (Badreddine et al., 2022). Notably, despite reduction in DMS activity during learning, D1 antagonism of the DMS did not improve coordination, suggesting that an initial re-organization of the neuronal circuit may be necessary for improvement in a specific skill, and disruption of this system (in this case by D1 antagonism) can alter the process. Further work should explore additional approaches that inhibit the dorsal striatum during and after training to evaluate the contribution of DLS more precisely in learning versus performance. The use of miniature microscopes or 2-photon imaging that image the activity of individual neurons may address the reorganization of neuronal clusters *in vivo* while the mouse is running on a treadmill.

Conversely, inhibition of DMS D1-SPNs did not decrease coordination scores after learning, consistent with the idea that later in training the DMS is disengaged and no longer necessary for expression of the skill (Badreddine et al., 2022; Cataldi et al., 2022). This is consistent with work in monkeys and rats, where dopamine transmission is required to learn reward-related behaviors, but is no longer required once the behavioral response is learned (Graybiel, 1998; Schultz et al., 2015; Schultz, 2019). Indeed, this step may be required to “chain” more and more complex behaviors. Other studies have demonstrated a dopamine-dependent change in synaptic plasticity that lasts for months and does not require dopamine to be present after it is established (Bamford et al., 2008). In summary, these results suggest that dopaminergic regulation of activity of D1-SPNs in the DMS is required for proper skill acquisition, while DLS direct pathway neurons may contribute to performance in early training, although at this juncture, a contribution to learning cannot be excluded.

Given the complexity of the circuits involved in motor learning and the multiple behavioral variables of each task, caution is required when interpreting the animal behavior and the interaction between the different brain regions. Dopamine neurons display different patterns of activation in different behavioral tasks, e.g., in head fixed versus freely moving animals or depending on whether a reward is present (Coddington and Dudman, 2019) and the activity of striatal neurons may be influenced by similar considerations (Cataldi et al., 2022). We note that the treadmill protocol could encompass aspects of aversion, as the mouse attempts to avoid hitting the back of the box, although we observed no change in calcium signals when the mouse was placed in the box, arguing against a classical fear response. A similar approach could be developed to examine spontaneous running on a wheel, which would avoid box confinement and the forced component of running given by the motorized system.

The Treadmill Training Task provides a means to evaluate motor ability in healthy animals and disease models and is designed to be useful for the evaluation of motor disorders and motor learning deficits because of neurodevelopmental disease, drug dependence, neurotoxic regimens, stroke, seizures, or injury.

## Synthesis

Reviewing Editor: Michael Michaelides, NIDA-NIH Decisions are customarily a result of the Reviewing Editor and the peer reviewers coming together and discussing their recommendations until a consensus is reached. When revisions are invited, a fact-based synthesis statement explaining their decision and outlining what is needed to prepare a revision will be listed below. The following reviewer(s) agreed to reveal their identity: Kari Johnson.

Reviewer 1

The authors investigate the role of direct pathway neurons and dopamine signaling at D1 receptors in the DMS and DLS in motor coordination learning. Here, that was assessed by recording D1 neuron activity with fiber photometry as mice learn to run on a treadmill at variable speeds. DLC based pose estimation was used to track head and paw position for computation of motor coordination variables. DMS, but not DLS calcium events decreased across training as running coordination improved. D1 receptor antagonism in the DLS and DMS impaired running coordination learning, while DLS antagonism also impaired coordination performance after learning.

These results are interesting and build on a growing literature investigating the role of DMS vs DLS in movement, skills, and learning. I have several comments on the data analysis, including potential ways that this dataset could be interrogated to better demonstrate the relationship between direct pathway activity and movement.

The features of the motor coordination score make sense to me, but it would be nice to have a little more context for these specific measures as the ones to focus on to quantify motor coordination. Is there some independent validation of the motor coordination score you use? Or a general background literature on gait/coordination assessments that would be cited here.

The authors show body weight split by sex, where there any sex differences in motor coordination score or other behavioral features?

How are calcium events detected? There are different approaches to this, what was done here is not described.

My main comments relate to the photometry analysis - A finer level analysis of the D1 neuron signal dynamics is called for here. We only see calcium “events” averaged over multi-minute periods, with no average signal traces aligned to any behavioral/movement variables. What is the relationship between ongoing behavior and D1 activity? There is a literature background of photometry and single cells imaging in the direct pathway that suggests in some cases rich encoding of movement and action sequences. These data can build on that previous work, but with the current limited analysis that’s not possible.

Related, calcium “events” averaged over a 5 minute window do not really tell us much about how the activity of these neurons relates to movement. Given the rich behavioral quantification, it would be great to see a more thorough investigation of the neural signals. It is also worth pointing out that there is disagreement over what exactly a calcium “event” means, which I think is another reason to go beyond such a reduced look at the photometry data. I don’t think you need to get rid of the event analysis, but adding additional measures, like signal correlations with movement onset, speed, etc, would increase the robustness here. Another thought would be to focus some analysis on the windows around when acceleration occurs, when you increase the treadmill speed.

In the results you say that you correlate brain activity with the motor coordination scores, but I am not seeing those analyses anywhere.

Separate 1-way ANOVAs on different treadmill sessions is not really the appropriate way to demonstrate that DMS or DLS signals are changing differentially over time. It would be better to do mixed effects ANOVAs and incorporate region, session, and repeated measures factors. Or at least within region repeated measures ANOVAs incorporating epoch (ON/OFF time) and session as factors.

I’m trying to reconcile the DMS photometry and D1 antagonism results. The authors argue that a decrease in DMS D1 activity is needed for the development of motor coordination - DMS calcium event amplitude goes down across training. At the same time, antagonizing D1 receptors impairs/delays normal motor coordination learning. One of the main effects of D1 antagonism would be to prevent dopamine from acting on D1 neurons, which should overall result in diminished D1 neuron activity. If reduced D1 activity in DMS is important for motor coordination, you might then expect that D1 receptor antagonism would promote faster motor coordination, rather than what is shown here. Some discussion of the role of dopamine versus D1 neurons themselves here is warranted.

Reviewer 2

The manuscript under review utilizes a custom-made treadmill task in combination with DeepLabCut analysis of mouse position/movement on the treadmill over the course of training to investigate the relationship between motor learning and activity of striatal direct pathway neurons. The investigators then use a pharmacological approach to assess the role of D1 receptor activation in motor learning. The behavioral task and analysis will likely be of broad interest and are a useful and affordable alternative to expensive commercial products that allow detailed analysis of gait. The utility of this resource is further enhanced by the authors providing easy access to their custom programs for the behavioral task and analysis. However, I am concerned that as analyzed and presented, the data do not adequately support the conclusions put forth by the authors.

1) The main claim of the paper, that DMS D1-SPN activity changes over the course of training whereas DLS D1-SPN activity does not, is not statistically analyzed in a way that directly tests these claims. To make the claim that DMS activity changes over the course of training, it is not appropriate to separately compare “on” and “off” activity at different time points and then claim that there’s a difference over time just because some of those individual comparisons are significant whereas others are not (Nieuwenhuis et al., Nature Neuroscience 2011 provides a helpful explanation of this). It’s also unclear why the comparison to “off” time is necessary rather than just looking at the amplitudes of events over the course of training; the authors should provide justification for this approach. The analysis presented for the peak # data in Fig. S2A is a more appropriate approach, and it’s unclear why the amplitude data were treated differently than the peak # (event rate) data. Further, it is unclear in all statistical analyses whether repeated/within-subject comparisons were treated as such in the statistical models. Clearer reporting of statistics including F values, degrees of freedom, exact p values, and within vs. between group factors would improve the transparency of the analyses. A direct statistical comparison of DMS vs. DLS activity change over time would also help support the claim that there is a training-related difference between the two regions.

2) The authors claim in the abstract and discussion that D1 receptor activation in the DLS is important for both initial learning and maintenance of learned motor coordination late in training, but based on the statistical analyses presented, the claim of a role for D1 in the DLS late in training does not appear to be substantiated by the data.

3) A key feature of the behavioral task is that the treadmill speed accelerates over the course of each session, yet the authors do not present any analyses that assesses whether neuronal activity is related to speed. This is particularly relevant given recent reports that various cell types in the striatum, including SPNs, encode speed during spontaneous activity (e.g., see Fobbs et al. J Neuro 2020).

4) The title of the paper overstates the findings, as the data do not establish a causal relationship between dopamine transmission and the observed training-dependent changes in DMS activity.

In addition, there are a number of smaller points that should be addressed:

1) The description of animal use is currently inadequate. At a minimum, additional information about light cycle, housing conditions (single or group housing?), and food/water access should be included.

2) In several cases, the placeholder “column factor” is used to present an experimental factor when two-way ANOVAs are reported. The authors should provide descriptive names for all factors included in their analyses.

3) Placement of optical fibers should be shown for all animals if possible (this could be included in supplemental figure 5). It would also be helpful for the sample micrographs to demonstrate GCaMP expression around the site of an optical fiber.

4) Repeated infusions of drug into brain tissue can cause damage. Did the investigators assess whether their repeated direct site infusions of the D1 antagonist (containing 1% DMSO) caused any local tissue damage? Was the DMSO concentration matched in saline controls?

5) In paragraph 2 of the results section, the authors state that “no difference in behavior or calcium signals was found between the 30 second periods before and after the running time” after describing a behavior validation experiment that did not seem to include fiber photometry. This should be clarified.

6) Although it’s wonderful to publish an affordable and open-source treadmill system, the discussion overstates the novelty of this approach, as commercial products (e.g., DigiGait by MouseSpecifics) have been available to perform this type of task along with detailed gait and coordination analysis for a while.

7) In several cases, data are presented on the same graph despite being statistically analyzed separately. This can be misleading at times.

8) Fig. S3 reports a 0.2 mg/kg dose of SCH39166, which sounds like a systemic injection and does not match with what is reported in the methods. Can this be clarified?

9) The discussion omits some important context related to prior studies of DMS vs. DLS roles in motor skill learning. For example, Kupferschmidt et al., Neuron 2017 showed a similar pattern of DMS disengagement vs. maintained DLS activity during rotarod learning when measuring the activity of presynaptic cortical inputs to these regions. Because cortical inputs to SPNs are known to be critical drivers of their activity, this is important context in conjunction with the findings in brain slice preparations that are currently included in the discussion (Badreddine et al., 2022).

## Author Response

Thank you for giving us the opportunity to submit a revised version of our article “A dopamine-dependent decrease in dorsomedial striatum direct pathway neuronal activity is required for learned motor coordination” to eNeuro.

We appreciate the time and effort that you and the reviewers have dedicated to providing valuable feedback on our manuscript. We are very grateful to the reviewers for their helpful comments and have been able to incorporate changes to reflect nearly all of their suggestions.

We detail the changes to the manuscript in this letter.

Comments by Referee 1

R: 1. The authors investigate the role of direct pathway neurons and dopamine signaling at D1 receptors in the DMS and DLS in motor coordination learning. Here, that was assessed by recording D1 neuron activity with fiber photometry as mice learn to run on a treadmill at variable speeds. DLC based pose estimation was used to track head and paw position for computation of motor coordination variables. DMS, but not DLS calcium events decreased across training as running coordination improved. D1 receptor antagonism in the DLS and DMS impaired running coordination learning, while DLS antagonism also impaired coordination performance after learning.

These results are interesting and build on a growing literature investigating the role of DMS vs DLS in movement, skills, and learning. I have several comments on the data analysis, including potential ways that this dataset could be interrogated to better demonstrate the relationship between direct pathway activity and movement.

The features of the motor coordination score make sense to me, but it would be nice to have a little more context for these specific measures as the ones to focus on to quantify motor coordination. Is there some independent validation of the motor coordination score you use? Or a general background literature on gait/coordination assessments that would be cited here.

A: We appreciate these comments and respond here and in the main text of the article.

We have developed a novel coordination score independently, and it is not based on previous evaluations, although, as Referee 2 mentions, similar commercial approaches (i.e., DigiGait) are available. We believe that our approach has important advantages over the commercial approach. Most importantly, our coordination score is based on multiple measures during treadmill running and we established that the combination of those features would better characterize an improvement in running ability: as we hope to make clear, each feature separately does not accurately represent the actual ability to run. This is because 1) Particularly at lower speeds, the mouse often explores the box while running. In this situation, a mouse may be rearing, grooming, moving sideways within the box. 2) At high speeds, the mouse is located more towards the back of the box, but is fully capable of running (the head position is low, but step length is appropriate). These factors would affect each individual components of the coordination score (step length, position of the head, direction of running).

For these reasons, we now include the point that the combination of these factors is more accurate in representing running capability. These points along with the analysis that led to the coordination score is now clarified in the main text and we have incorporated Referee 1’s suggestions throughout the manuscript. Additional sentences or modified content can be found in the Methods and in the main text in line 264 on page 12 and line 333 on page 15.

R: 2. The authors show body weight split by sex, where there any sex differences in motor coordination score or other behavioral features?

A: All the treadmill data analyzed by sex is now included and shown in Figure 1-1C. We did not find any difference between males and females in motor coordination or other tests, and thus combine the behavioral data for the main figure into one graph (shown in Fig 1 and Figure 1-1).

R: 3. How are calcium events detected? There are different approaches to this, what was done here is not described.

A: We now include a more detailed description of the analysis process in the Methods. (Section Calcium signal analysis). The code for the analysis will also be available on our github page for everyone to access, and it is also attached as supplementary material.

R: 4. My main comments relate to the photometry analysis - A finer level analysis of the D1 neuron signal dynamics is called for here. We only see calcium “events” averaged over multi-minute periods, with no average signal traces aligned to any behavioral/movement variables. What is the relationship between ongoing behavior and D1 activity? There is a literature background of photometry and single cells imaging in the direct pathway that suggests in some cases rich encoding of movement and action sequences. These data can build on that previous work, but with the current limited analysis that’s not possible.

Related, calcium “events” averaged over a 5 minute window do not really tell us much about how the activity of these neurons relates to movement. Given the rich behavioral quantification, it would be great to see a more thorough investigation of the neural signals. It is also worth pointing out that there is disagreement over what exactly a calcium “event” means, which I think is another reason to go beyond such a reduced look at the photometry data. I don’t think you need to get rid of the event analysis, but adding additional measures, like signal correlations with movement onset, speed, etc, would increase the robustness here.

A: In agreement with this critique, we have extended our analysis of calcium and behavioral data to include evaluation of single behavioral variables and calcium epochs. Data is now discussed in the main text in line 333 page 15 and included in Figure 2-2. The data did not show any apparent peak activity at the time of individual variables for any of the animals (only sample animals are shown in the figure). Please note that our approach likely detects a variety of signals that may be independent of a specific behavior variable. Further experiments with more refined techniques (2-photon or miniature microscopes) may be able to address this in more detail. We further elaborate on this in line 428 on page 19.

The new code we have written to perform the analysis is included in the method section and our github page and may be of general help for the field.

R: 5. Another thought would be to focus some analysis on the windows around when acceleration occurs, when you increase the treadmill speed.

A: We now include this analysis in Figure 2-2. The data are not significant. We mention this in the main text in line 339 and page 15, and the code used for the analysis is likewise included on the github page.

R: 6. In the results you say that you correlate brain activity with the motor coordination scores, but I am not seeing those analyses anywhere.

A: We agree with the reviewer and have adjusted the main text accordingly. See line 10 and 14 page 1, line 72 page 3, and line 299 page 13.

R: 7. Separate 1-way ANOVAs on different treadmill sessions is not really the appropriate way to demonstrate that DMS or DLS signals are changing differentially over time. It would be better to do mixed effects ANOVAs and incorporate region, session, and repeated measures factors. Or at least within region repeated measures ANOVAs incorporating epoch (ON/OFF time) and session as factors.

A: We agree with the Referee’s recommendations and now include additional graphs in Figure 2-1. These include the plot of calcium signals across all 12 days comparing ON and OFF times within regions (Figure 2C and D) with home cage baseline. The figure from home cage baseline has now been incorporated into Figure 2-1C and D and the main text modified accordingly (line 290 on page 13). Statistics for these figures are also included in the main text in line 313 on page 14. Epoch counts are now moved to Figure 2-1E and F.

R: 8. I’m trying to reconcile the DMS photometry and D1 antagonism results. The authors argue that a decrease in DMS D1 activity is needed for the development of motor coordination - DMS calcium event amplitude goes down across training. At the same time, antagonizing D1 receptors impairs/delays normal motor coordination learning. One of the main effects of D1 antagonism would be to prevent dopamine from acting on D1 neurons, which should overall result in diminished D1 neuron activity. If reduced D1 activity in DMS is important for motor coordination, you might then expect that D1 receptor antagonism would promote faster motor coordination, rather than what is shown here. Some discussion of the role of dopamine versus D1 neurons themselves here is warranted.

A: This critique is very important, and we have tried to address it clearly in this revision. The D1 antagonists indeed decrease SPN activity (please see Figure 3-1), and we have made changes throughout the manuscript to better describe this role based on previous literature and summarize our results to better suggest how D1 receptor activation is required for adequate circuit refinement.

Specifically, we suggest that an initial level of SPN and DA neuron activity in naïve animals is necessary for skill acquisition. Then, with practice as the skill is refined, the DMS may disengage from the action, which is consistent with fewer neurons required for the specific task. Blockade of the D1 activity during the first phase, when the activity is necessary, will affect this refinement. In contrast, after the skill is learned in tandem with the synaptic refinement, D1-antagonism no longer affects the task.

Notably, these observations on a role for dopamine in initiating experience-dependent plasticity, but not being required to maintain it, is consistent with many studies of dopamine in reward based behaviors, including the well known “reward prediction error” studies of Wolfram Schultz (https://pubmed.ncbi.nlm.nih.gov/31588354/) and Ann Graybiel (https://pubmed.ncbi.nlm.nih.gov/9753592/) in monkeys, and Mark Wightman and Regina Carelli (https://pubmed.ncbi.nlm.nih.gov/26719853/) in rats, in which dopamine transmission is required to learn a behavioral response, but is no longer required after the response is learned: indeed, this step is probably required to “chain” more and more complex behavior. Our lab’s work has also demonstrated a dopamine dependent change in synaptic plasticity that lasts for months and not only does not require dopamine to be present after it is established, but dopamine can return the synapses to a state like the naïve condition even months later (https://pubmed.ncbi.nlm.nih.gov/18400166/). Thus, there is a literature that we now discuss that is consistent with a requirement for dopamine in the establishment of forms of synaptic plasticity that do not require dopamine once established. We have attempted to provide more clear commentary in the main text in line 423 and 433 on page 19.

Comments by Referee 2

R:The manuscript under review utilizes a custom-made treadmill task in combination with DeepLabCut analysis of mouse position/movement on the treadmill over the course of training to investigate the relationship between motor learning and activity of striatal direct pathway neurons. The investigators then use a pharmacological approach to assess the role of D1 receptor activation in motor learning. The behavioral task and analysis will likely be of broad interest and are a useful and affordable alternative to expensive commercial products that allow detailed analysis of gait. The utility of this resource is further enhanced by the authors providing easy access to their custom programs for the behavioral task and analysis. However, I am concerned that as analyzed and presented, the data do not adequately support the conclusions put forth by the authors.

R: 1. The main claim of the paper, that DMS D1-SPN activity changes over the course of training whereas DLS D1-SPN activity does not, is not statistically analyzed in a way that directly tests these claims. To make the claim that DMS activity changes over the course of training, it is not appropriate to separately compare “on” and “off” activity at different time points and then claim that there’s a difference over time just because some of those individual comparisons are significant whereas others are not (Nieuwenhuis et al., Nature Neuroscience 2011 provides a helpful explanation of this). It’s also unclear why the comparison to “off” time is necessary rather than just looking at the amplitudes of events over the course of training; the authors should provide justification for this approach. The analysis presented for the peak # data in Fig. S2A is a more appropriate approach, and it’s unclear why the amplitude data were treated differently than the peak # (event rate) data. Further, it is unclear in all statistical analyses whether repeated/within-subject comparisons were treated as such in the statistical models. Clearer reporting of statistics including F values, degrees of freedom, exact p values, and within vs. between group factors would improve the transparency of the analyses. A direct statistical comparison of DMS vs. DLS activity change over time would also help support the claim that there is a training-related difference between the two regions.

A: We appreciate this critique and note that Referee 1 also suggested a similar statistical approach. We have now corrected Fig 2-1 to include this data with the analysis suggested by both referees (Please see reply to point 6 by Referee 1).

Given that DMS and DLS activities were recorded from different mice and are expected to possess different levels of signal/viral expression, we do not think it is helpful to directly compare the levels in the two groups statistically: specifically, the signal in the DLS is generally smaller and that difference in overall signal levels between the two region is not dependent on training. Rather, we think it more reliable to compare the change in signals in both regions over training and the course of this change is different between the two regions. We hope that the additional graph and statistics included will address this concern. In particular, we include Supplementary Figure 2 C and D where changes in calcium peak amplitudes during running are plot together with home cage baseline recording and off time (time in which the treadmill is not moving). Statistically, we analyzed each group individually and identified changes in the signal over time, rather than across groups. The comparison across groups is still shown in Figure 2 C and D, although the figure now includes home cage baseline recordings for day 1, 3, 7, and 12. We have detailed the additional statistical procedures in the main text and in corresponding figures. This can be found in line 292 on page 13 and 303 on page 14, as well as in Figure 2 and Figure 2-1.

R: 2. The authors claim in the abstract and discussion that D1 receptor activation in the DLS is important for both initial learning and maintenance of learned motor coordination late in training, but based on the statistical analyses presented, the claim of a role for D1 in the DLS late in training does not appear to be substantiated by the data.

A: We agree with this important point, the data on maintenance did not reach significance and so does not show a difference between the two groups. We removed this suggestion from the paper. Indeed, as we detail in reply to Reviewer 1, point 7, dopamine may not be required to maintain synaptic changes. We have rewritten the abstract and adjusted the main text accordingly in line 76 on page 4, line 414 on page 19 and 442 on page 20.

R: 3. A key feature of the behavioral task is that the treadmill speed accelerates over the course of each session, yet the authors do not present any analyses that assesses whether neuronal activity is related to speed. This is particularly relevant given recent reports that various cell types in the striatum, including SPNs, encode speed during spontaneous activity (e.g., see Fobbs et al. J Neuro 2020).

A: We agree, and Referee 1 made a similar comment about acceleration. We now include Fig 2-2, which shows no obvious connection between time of acceleration of the treadmill and calcium signals. The referee is correct by citing studies in which mouse velocity related to specific activity in SPNs, but note that in those studies, investigators recorded activity during spontaneous locomotion in an open field or with head fixed running on a treadmill, and examined specific features of the mouse movement at different resolution than in our study. We performed a similar type of analysis (see above, Referee 1, point 3), but found no relationship between individual peaks and velocity. This may be because our coordination learning task entails running triggered by the investigator rather than the mouse spontaneously initiating running. We elaborate this further in line 339 page 15.

R: 4. The title of the paper overstates the findings, as the data do not establish a causal relationship between dopamine transmission and the observed training-dependent changes in DMS activity.

A: We appreciate this point and have rewritten the title as “Decrease in dorsomedial striatum direct pathway neuronal activity is required for learned motor coordination"

R: In addition, there are a number of smaller points that should be addressed: 1) The description of animal use is currently inadequate. At a minimum, additional information about light cycle, housing conditions (single or group housing?), and food/water access should be included.

A: We thank Referee 2 for noticing this and now include this information in the method section (line 87 on page 4).

R: 2) In several cases, the placeholder “column factor” is used to present an experimental factor when two-way ANOVAs are reported. The authors should provide descriptive names for all factors included in their analyses.

A: This is an important comment. The factors are days and condition (time running, time off, and baseline). We now include the factors in all statistical measurements and in the corresponding figures for each plot in the article.

R: 3) Placement of optical fibers should be shown for all animals if possible (this could be included in supplemental figure 5). It would also be helpful for the sample micrographs to demonstrate GCaMP expression around the site of an optical fiber.

A: We now include additional figures showing placement of the fibers as well as a schematic of location of all fibers to Figure 2-3 (previously Supplementary Figure 5).

R: 4) Repeated infusions of drug into brain tissue can cause damage. Did the investigators assess whether their repeated direct site infusions of the D1 antagonist (containing 1% DMSO) caused any local tissue damage? Was the DMSO concentration matched in saline controls?

A: We agree with this concern on performing local infusions. We found that repetitive infusion of the drug did not cause visible damage (as shown in Figure 2-3), nor increased mortality or noticeably altered behavior in injected mice (personal observation, data not shown). Saline was prepared with the same concentration of DMSO for injections in control mice, and delivered in the same amounts and procedure. This is now stated in the methods section “Pharmacological experiments”.

R: 5) In paragraph 2 of the results section, the authors state that “no difference in behavior or calcium signals was found between the 30 second periods before and after the running time” after describing a behavior validation experiment that did not seem to include fiber photometry. This should be clarified.

A: We apologize for the error and have now moved the sentence to the correct section (see line 300 page 14) .

R: 6) Although it’s wonderful to publish an affordable and open-source treadmill system, the discussion overstates the novelty of this approach, as commercial products (e.g., DigiGait by MouseSpecifics) have been available to perform this type of task along with detailed gait and coordination analysis for a while.

A: We appreciate this comment and commercial products are indeed available. We argue that the openness of our model makes it a more powerful tool as it can be adjusted more easily to one’s needs, as discussed in our reply to Reviewer 1, comment 1 on the calculation of the coordination score. We removed the sentence “To our knowledge, this is the first paradigm to measure changes in the activity of identified neurons during the learning of a complex and extended task requiring improvements in motor coordination” from the Discussion.

R: 7) In several cases, data are presented on the same graph despite being statistically analyzed separately. This can be misleading at times.

A: Thank you for this critique. This relates to previous comments from both referees (see point 1 from referee 2 and point 6 from referee 1). We now include additional plots and statistical analysis accordingly (Figure 2-1).

R: 8) Fig. S3 reports a 0.2 mg/kg dose of SCH39166, which sounds like a systemic injection and does not match with what is reported in the methods. Can this be clarified?

A: Thank you for noting this issue. The dose chosen for this study is the same as systemic injections (0.2 mg/kg), as we wanted a substantial effect. This is now clarified in the methods. The dosage shown in the methods now matches that of Fig 3-1.

R: 9) The discussion omits some important context related to prior studies of DMS vs. DLS roles in motor skill learning. For example, Kupferschmidt et al., Neuron 2017 showed a similar pattern of DMS disengagement vs. maintained DLS activity during rotarod learning when measuring the activity of presynaptic cortical inputs to these regions. Because cortical inputs to SPNs are known to be critical drivers of their activity, this is important context in conjunction with the findings in brain slice preparations that are currently included in the discussion (Badreddine et al., 2022).

A: This is an excellent point, and we now include additional references to the study (Kumpferschmidt et al. 2017), and sentences in line 389 page 17 to support this evidence for alterations in the presynaptic cortical inputs during learning.

Additional modifications:

The reference list in the manuscript have been updated to include the additional references mentioned in our response to the referees. Figures have also been updated accordingly.

Siham Boumhaouad was included in the author list for her contribution to imaging and analysis.

We are grateful to the Editor and Reviewers for these extraordinarily useful and detailed critiques, which we think have vastly improved the manuscript. We look forward to responding to any further questions and comments.
