# Supplementary figures and images for: Decreased Dorsomedial Striatum Direct Pathway Neuronal Activity Is Required for Learned Motor Coordination
Source: eNeuro. 2022 Oct 10;9(5):ENEURO.0169-22.2022. doi: 10.1523/ENEURO.0169-22.2022 (PMC9557335; doi:10.1523/ENEURO.0169-22.2022)

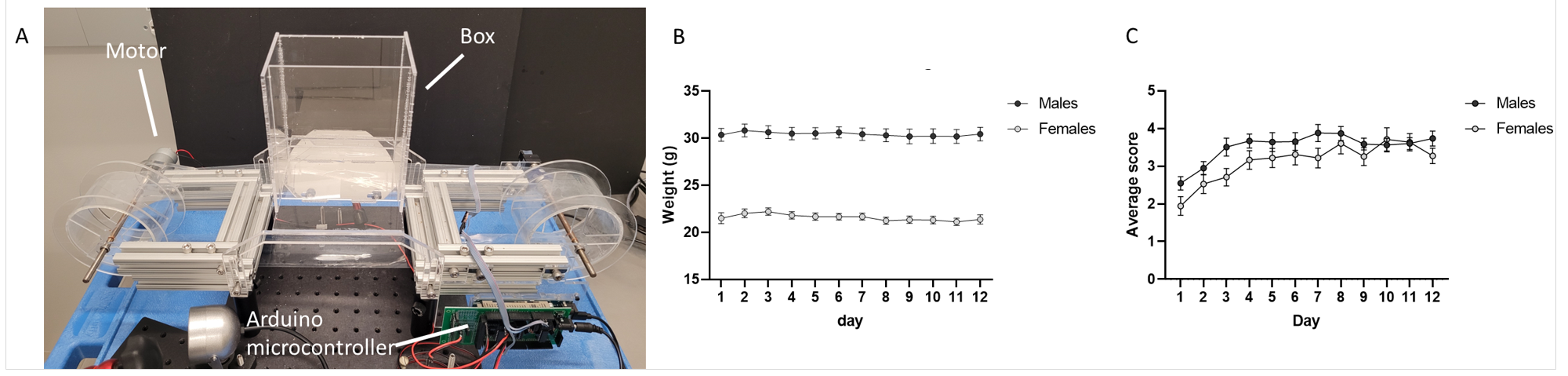

Supplement: Extended Data Figure 1-1 — A, The custom-built treadmill system. A clear circular belt is moved by a motor controlled by a microcontroller. A clear acrylic box is placed around the running area to prevent the mouse from jumping off the treadmill during the experiment. A mirror is placed below the treadmill to visualize movement of all four paws during running via a camera located on the side (not in picture). B, Mouse weight was recorded before each day of experiment; 12 consecutive days of training did not affect mouse weight (one-way ANOVA p = 0.90 for time). Data showing differences between male and female weight at this age (3–4 months of age; n = 11 males; n = 7 females; two-way ANOVA p < 0.0001). C, Motor coordination score for all cohorts by sex. Male and female animals show similar significant improvement in the coordination score over the 12 d of testing but without any differences between the two sexes (males n = 15 and females n = 11, two-way ANOVA Day vs Sex p = 0.33, Day ****p < 0.0001, Sex p = 0.07, Subject ****p < 0.0001). Download Figure 1-1, TIF file. [file enu-eN-OTM-0169-22-s03.tif]

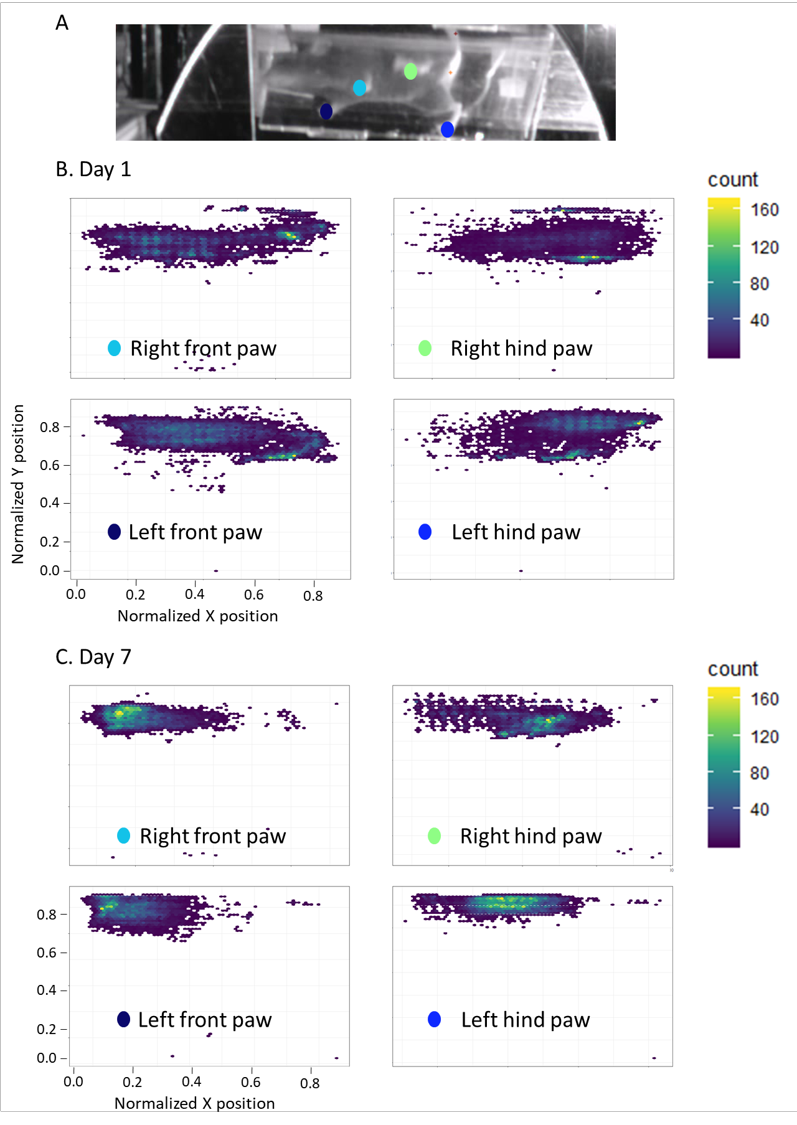

Supplement: Extended Data Figure 1-2 — Heatmaps of paw placement data obtained from DeepLabCut for one mouse on day 1 (B) and day 7 (C) of the Treadmill Training Task. Each heatmap represents data from a different paw and is color labeled as per the schematic in A and in Figure 1B (top left for the right front paw, light blue dot in panel A; bottom left for the left front paw, dark blue dot in panel A; top right for the right hind paw, green dot in panel A; bottom right for the left hind paw, blue dot for panel A). x- and y-axes values obtained from tracking are normalized. Each heat map shows likelihood of placing a specific paw in a specific set of coordinates. The blue color represents regions with low number of paw placements, while yellow indicates high chance of placing in those coordinates. B, Heatmaps for one control mouse on day 1 of training. The spread of paw placement indicates poor ability of the mouse to run into place. While running the mouse was placing its paws all over the 2D field and rarely in the same spot. C, Heatmaps for the same control animal on day 7 of training. This data show more consistent paw placement with the mouse stepping mostly in the same area. This shows the ability of the mouse to run in place, indicating improved proficiency in running. Download Figure 1-2, TIF file. [file enu-eN-OTM-0169-22-s06.tif]

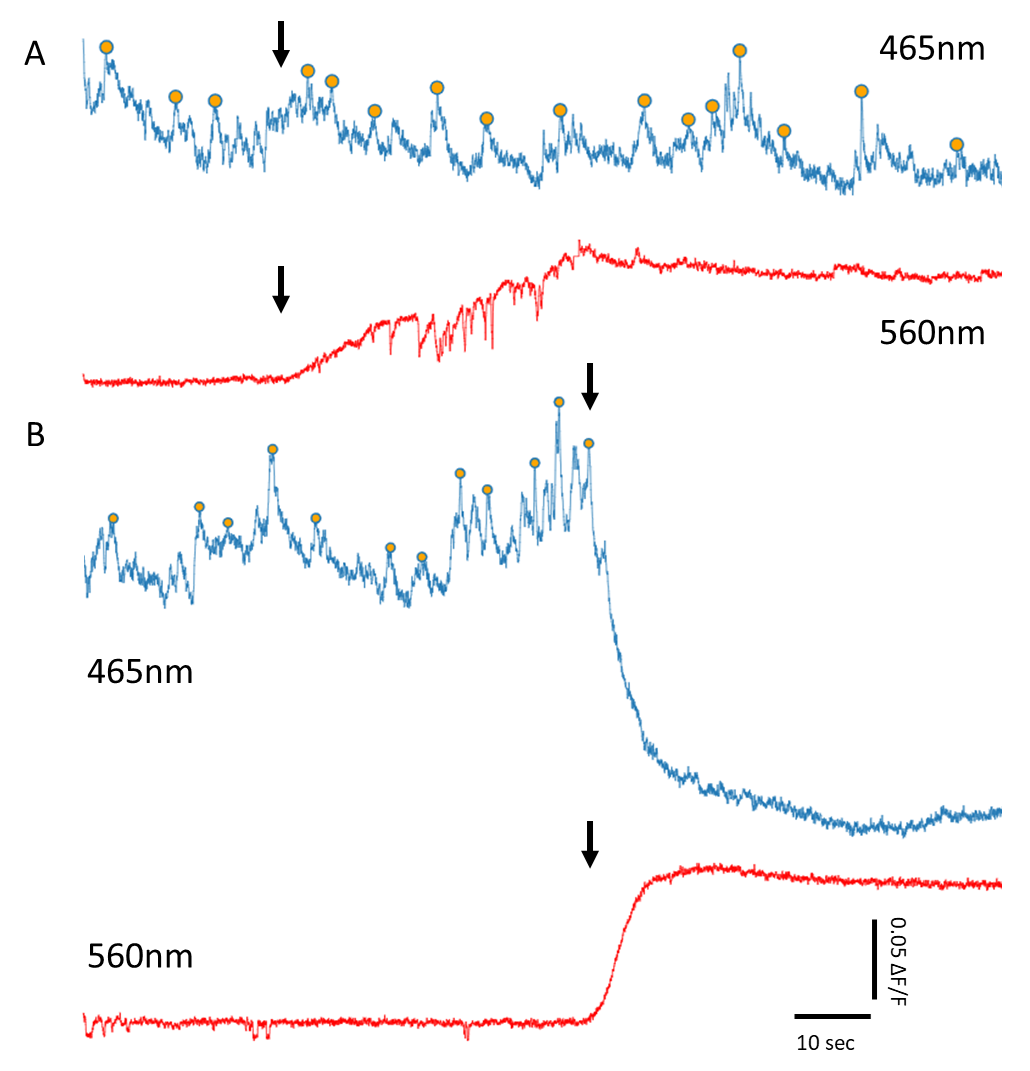

Supplement: Extended Data Figure 3-1 — A, Representative calcium trace (in blue, detected events indicated by orange dots) of D1-SPNs GCaMP6f signal in the DMS of a freely moving mouse, while injected with a control solution of saline + rhodamine. Rhodamine signal is recorded at 560 nm to confirm injection local to the photometry fiber (red). Local injection did not alter calcium signals in this control. Injection time indicated by arrows. B, Similar recording of D1-SPNs in the DMS during injection of a D1-antagonist (SCH39166, 48.13 µm), in solution with rhodamine. Injection of the antagonist significantly reduced the baseline calcium signal and eliminated calcium events in the DMS. Download Figure 3-1, TIF file. [file enu-eN-OTM-0169-22-s05.tif]

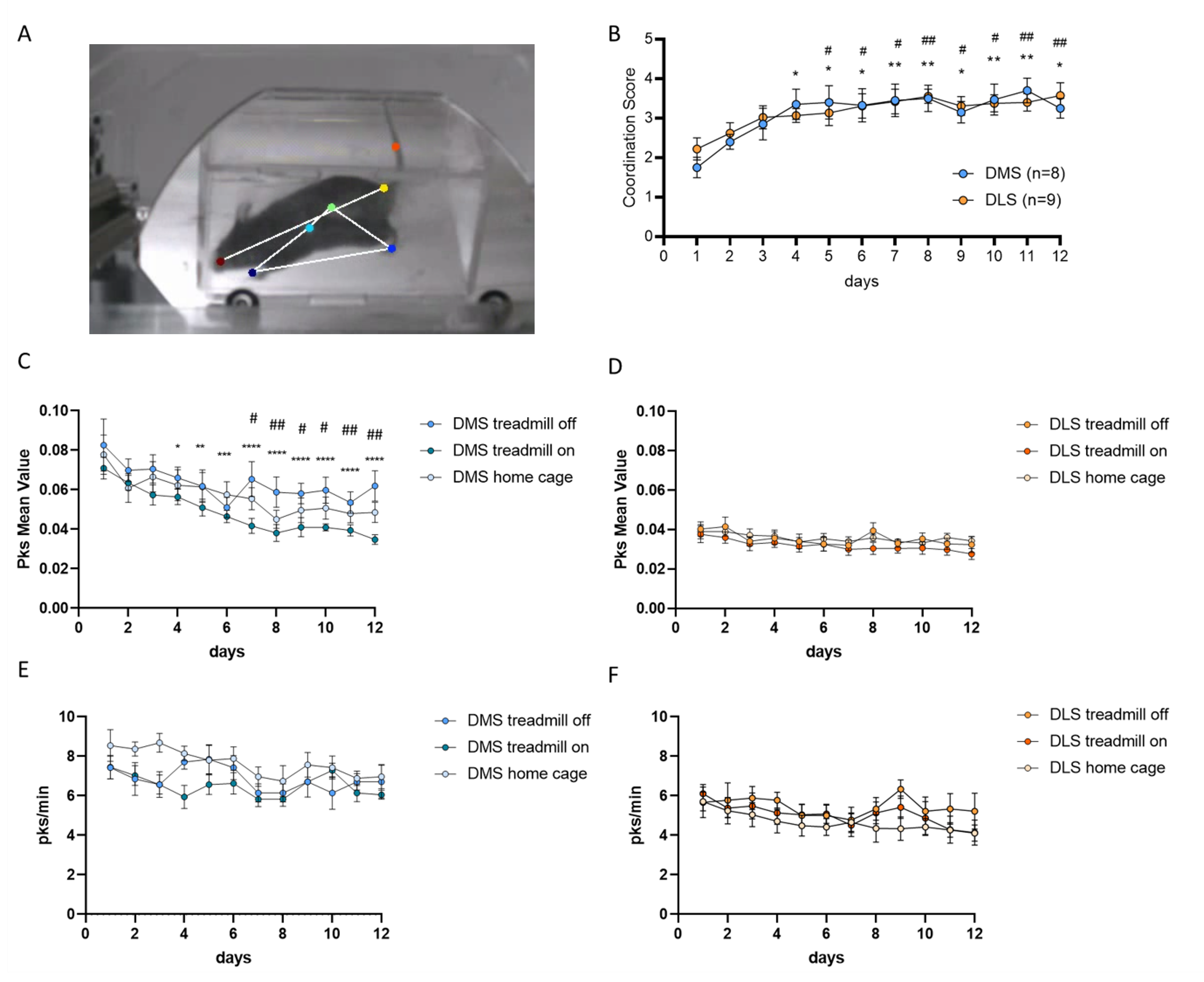

Supplement: Extended Data Figure 2-1 — A, Sample image from DeepLabCut as the mouse is running on the treadmill. Each paw, the head, the base of the tail, and the tail are labeled by colored dots. B, Coordination score from treadmill running for DMS-injected and DLS-injected animals. Mice performed significantly better within 2–4 d from day 1 of training, indicating that surgery, viral expression, and optic fiber implant did not affect running abilities and motor learning (one-way ANOVA **p < 0.01 for DMS mice and *p < 0.05 for DLS mice). C, D, Average calcium event peak amplitudes for D1-SPNs in the DMS (C) and DLS (D) over the 12 d of training. Peak epoch amplitudes are compared with the baseline home cage recording. There is no significant change in home cage baseline (one-way ANOVA p = 0.13 for DMS and p = 0.73 for DLS) and time-off (one-way ANOVA p = 0.21 for DMS and p = 0.50 for DLS). Average peak amplitude for DMS D1-SPNs significantly decreased during running time (one-way ANOVA p < 0.0001 for on-time alone, * represents post hoc significant values, and RM two-way ANOVA p < 0.01 when comparing on-time to off-time, # represents post hoc significant values; detailed statistics in the main text) although peak amplitudes average levels remained unchanged for DLS D1-SPNs (one-way ANOVA p = 0.64). E, F, Average calcium event rate for D1-SPNs in the DMS (E) and DLS (F). Measurements for epochs of running time (treadmill on), time in which the treadmill is off, and event counts in home cage baseline recording were overlapped. There were no significant changes in the average event rate over the 12 d of training for DMS (one-way ANOVA p = 0.08 for home cage baseline recording, p = 0.07 for running time, and p = 0.31) or DLS recording (one-way ANOVA p = 0.67 for home cage baseline recording, p = 0.59 for running time, and p = 0.93). Download Figure 2-1, TIF file. [file enu-eN-OTM-0169-22-s04.tif]

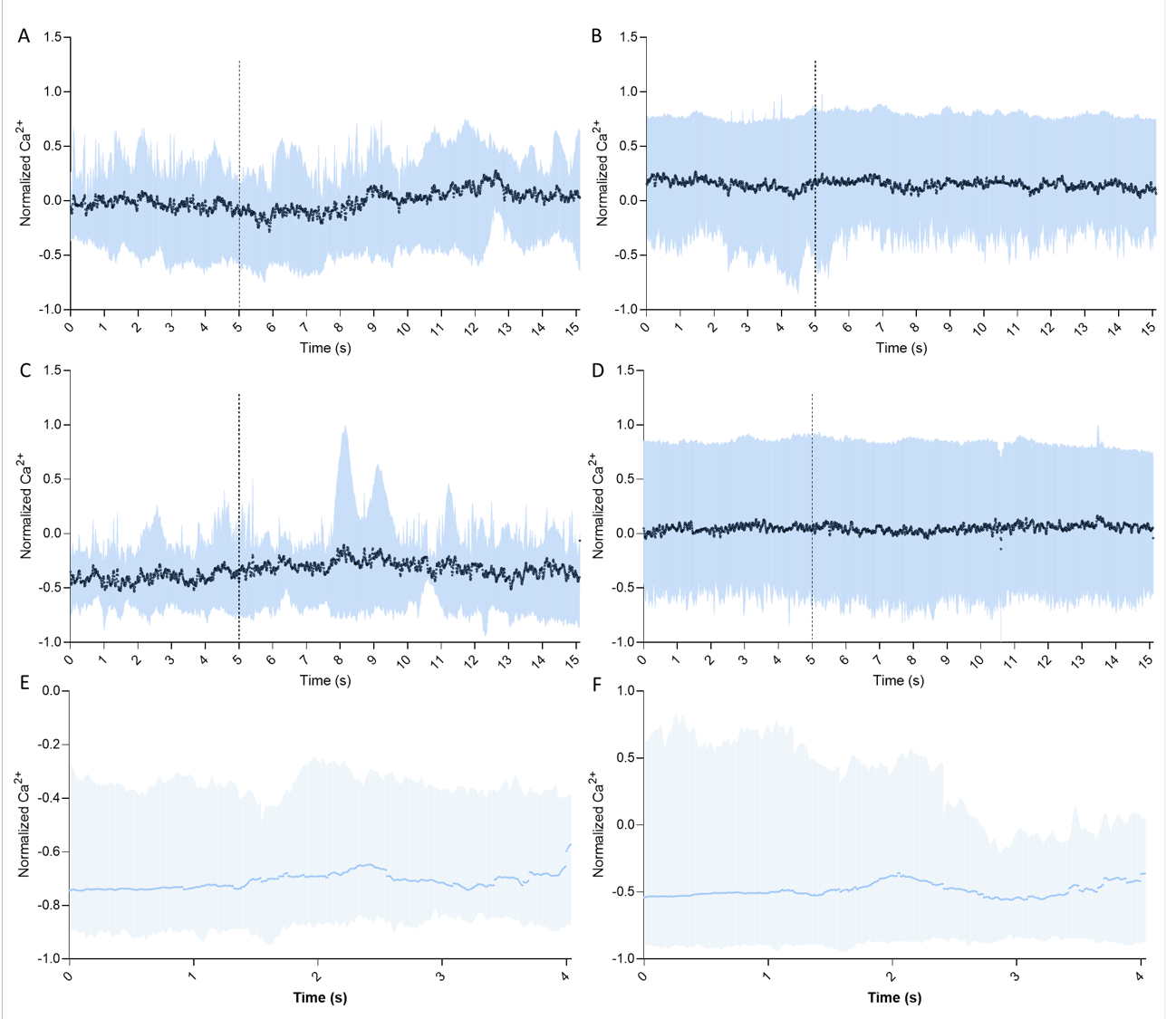

Supplement: Extended Data Figure 2-2 — A, B, Sample average calcium traces from D1-SPNs in the DMS of one mouse at the time of which the treadmill is turned on (indicated by dashed black line) for day 1 (A) and for day 12 (B). There is no apparent increase in calcium activity at that time. C, D, Similarly, there is no obvious increase in activity at the time in which the treadmill switches to the highest speed (10 m/min) for both day 1 (C) and day 12 (D; time of acceleration indicated by dashed black line). E, F, Sample average calcium traces for the DMS of one mouse at the time of significant events [high variation in mouse head position (E) or in step length (F)]. Mouse head position and step length were analyzed to detect moments during running in which the value significantly differs from the average. Corresponding calcium traces were identified and averaged together. No significant peak in calcium activity was detected during these events. Download Figure 2-2, TIF file. [file enu-eN-OTM-0169-22-s07.tif]

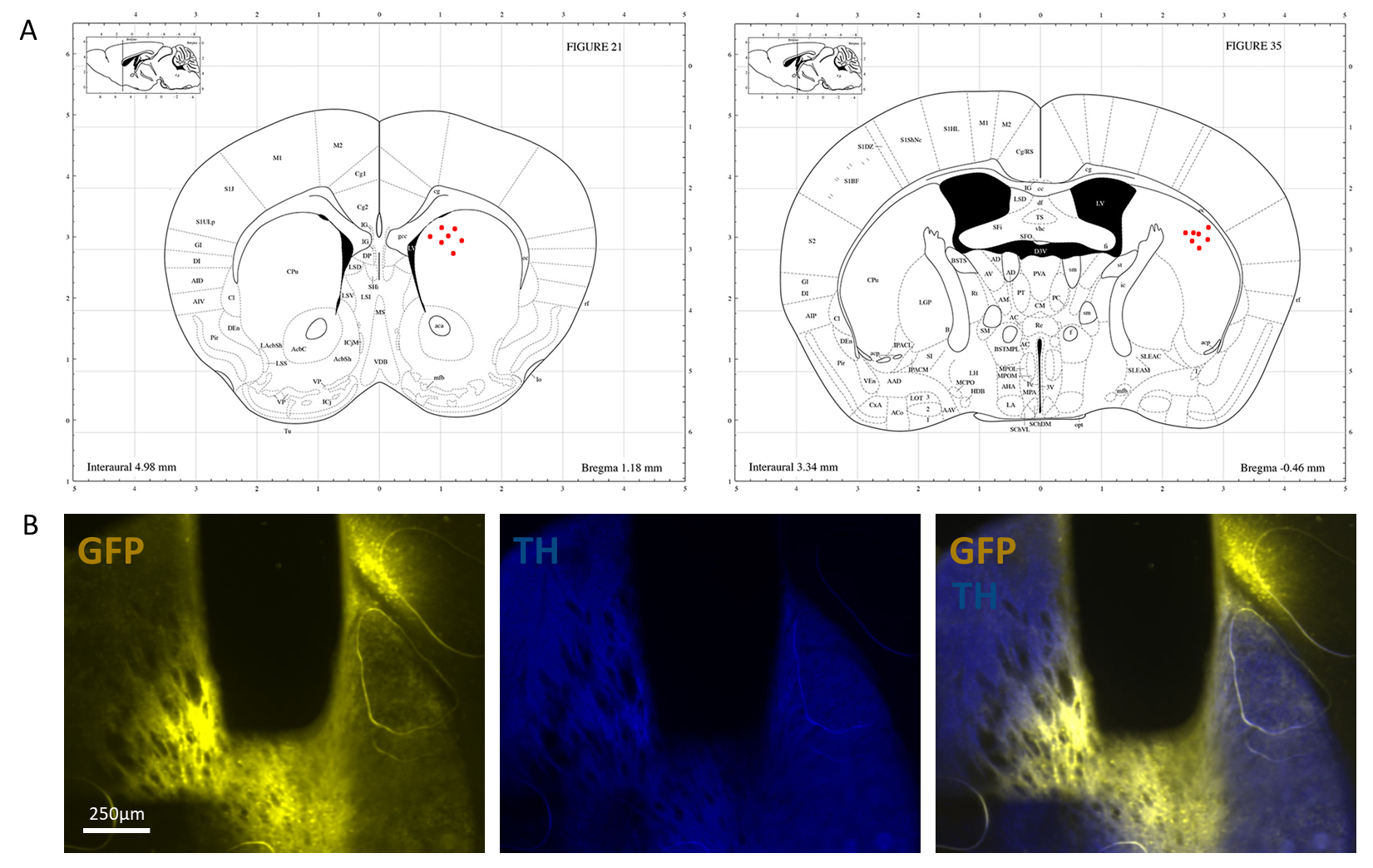

Supplement: Extended Data Figure 2-3 — A, Schematic of location of optic fibers for mice implanted in the DMS (left) and the DLS (right). B, Sample micrographs of coronal sections of the dorsal striatum of D1-cre animals injected with AAV vectors containing Flex-GCaMP6f. GCaMP6f expression is confirmed by GFP immunolabeling (yellow) co-stained with TH antibody (blue) to visualize the striatum. Download Figure 2-3, TIF file. [file enu-eN-OTM-0169-22-s08.tif]
